# Single-cell transcriptome-wide Mendelian randomization identifies mitochondrial targets in immune cells for Major Depressive Disorder

**DOI:** 10.3389/fimmu.2025.1700604

**Published:** 2026-01-12

**Authors:** Yangyi Zhang, Fei Zhang, Xingyi Shen, Maoyuan Ni, Jiayi Zhang, Shunling Zhang, Jingwen Lin, Zhaoyang Yang, Huangwei Lei

**Affiliations:** 1 Guangzhou University of Chinese Medicine, Guangzhou, Guangdong, China; 2 Fujian University of Traditional Chinese Medicine, Fuzhou, Fujian, China; 3 School of Rehabilitation Medicine, Gannan Medical University, Ganzhou, Jiangxi, China

**Keywords:** immune cell eQTL, machine learning, Major Depressive Disorder, Mendelian randomization, mitochondrial energy metabolism

## Abstract

**Background:**

A critical need exists for objective biomarkers and novel therapeutic targets in major depressive disorder (MDD). Although dysfunction in mitochondrial immunometabolism is implicated in MDD, the specific causal genes suitable for clinical translation remain largely unidentified. This study aimed to bridge this gap by identifying mitochondria-related genes that have a causal impact on MDD risk through their expression in specific immune cells.

**Methods:**

We integrated multi-omics data with machine learning to pinpoint key mitochondria-related energy metabolism genes (MEMRGs) linked to immune cell infiltration, assessed via ssGSEA and CIBERSORT algorithms. Cell-type-specific two-sample Mendelian randomization (MR) was employed to evaluate causal relationships between gene expression and MDD risk. Findings were validated in a chronic unpredictable mild stress (CUMS) rat model.

**Results:**

Our analysis identified five genes—*HK2, NDUFS4, NEU1, SOD1*, and *UCP2*—whose expression in distinct immune populations had significant causal effects on MDD risk. Notably, *HK2, NDUFS4*, and *NEU1* were identified as protective factors, while *UCP2* and *SOD1* were risk factors in specific cell types. The clinical relevance of this panel was supported by its diagnostic performance in an independent cohort, and the upregulation of the principal risk gene, *UCP2*, was confirmed in the hippocampus of CUMS rats.

**Conclusion:**

This study provides robust genetic evidence establishing a causal link between the expression of specific mitochondrial genes in immune cells and the risk of MDD. By prioritizing *UCP2, SOD1, HK2, NDUFS4*, and *NEU1*, our findings highlight novel, immune-mediated pathways in depression and nominate promising targets for future diagnosis and therapeutic intervention.

## 1 Introduction

Major Depressive Disorder (MDD) is a prevalent mental illness, characterized by persistent low mood, diminished self-esteem, loss of interest in activities, reduced concentration, self-harm, and suicidal tendencies (1, 2). Its global prevalence imposes a substantial socioeconomic burden, particularly as it often manifests between ages 20 and 30, affecting quality of life and contributing to high suicide rates (3, 4). Diagnosis remains reliant on subjective symptom criteria, leading to potential misdiagnosis, and a substantial portion of patients fail to respond to existing antidepressant therapies (5, 6). Identifying reliable biomarkers and underlying mechanisms is thus imperative for early detection, precision diagnostics, and targeted therapies to mitigate suicide. Emerging artificial intelligence tools, such as machine learning for multi-omics integration, show promise in objective MDD detection by enhancing biomarker accuracy (7, 8), yet causal biological pathways remain underexplored.

A growing body of evidence implicates immune dysregulation and neuroinflammation as central pillars in the pathophysiology of MDD (9, 10). The function, differentiation, and activation of key immune cells, such as T cells and monocytes, are intrinsically linked to their metabolic state, with mitochondrial energy metabolism serving as a critical regulatory hub (11, 12). While mitochondrial dysfunction in the brain has been independently associated with MDD (13, 14), its specific role within the immune system’s contribution to depression remains a critical knowledge gap. Identifying the specific mitochondrial energy metabolism-related genes (MEMRGs) that drive immune cell-mediated risk for MDD could therefore uncover a new class of therapeutic targets at the interface of metabolism and immunity.

Addressing this gap could uncover novel pathways amenable to clinical intervention. To bridge this knowledge deficit, we integrated multi-omics data from the Gene Expression Omnibus (GEO) database and GeneCards to identify mitochondrial energy metabolism-related differentially expressed genes (MEMRDEGs) in MDD. Key genes were selected using LASSO, support vector machine (SVM), and logistic regression analyses. Their diagnostic potential was evaluated via receiver operating characteristic (ROC) curves. Immune infiltration profiles were assessed using ssGSEA and CIBERSORT algorithms with dual validation, and correlations between gene expression and immune cell abundance were analyzed. To formally test for causality between gene expression in specific immune lineages and MDD pathogenesis, we innovatively implemented a stratified Mendelian randomization (MR) framework. By leveraging summary statistics from major MDD GWAS and our curated single-cell eQTL (sc-eQTL) data across 14 immune cell types as genetic instruments, we systematically assessed the causal impact of the identified key genes. Therefore, the primary aim of this study was to identify MEMRGs that causally modulate MDD risk via their expression in distinct immune cell populations. We hypothesized that this high-resolution approach would reveal a panel of novel, cell-type-specific biomarkers and prioritize actionable targets for the future development of immunomodulatory therapies for MDD.

## 2 Materials and methods

### 2.1 Data acquisition and preprocessing

We downloaded two MDD-related datasets, GSE98793 (15) and GSE76826 (16), from the GEO database (17) using the R package *GEOquery* (18). Both datasets were derived from human blood samples and provided gene expression profiles. The GSE98793 dataset, generated on the GPL570 [HG-U133_Plus_2] Affymetrix Human Genome U133 Plus 2.0 Array platform, included 192 samples (128 from MDD patients and 64 from healthy controls). The GSE76826 dataset, generated on the GPL17077 Agilent-039494 SurePrint G3 Human GE v2 8x60K Microarray 039381 platform, comprised 32 samples (12 from MDD patients and 20 from healthy controls). All samples were used in subsequent analyses. Detailed dataset information is provided in **Table 1**.

**Table 1:** MDD dataset information list.

Characteristics	GSE98793	GSE76826
Platform	GPL570	GPL17077
Species	Homo sapiens	Homo sapiens
Tissue	whole blood	blood
Samples in Normal group	64	12
Samples in MDD group	128	20
Reference	(15)	(16)

MEMRGs were obtained from the GeneCards database (19) using the keyword “Mitochondrial energy metabolism” and restricting to “Protein Coding” genes, yielding 218 MEMRGs. Additionally, we searched the Molecular Signatures Database (20) with the same keyword and identified 53 MEMRGs from the “KEGG_AMYOTROPHIC_LATERAL_SCLEROSIS_ALS” gene set. After merging and removing duplicates, a total of 266 unique MEMRGs were obtained. Detailed information is presented in **Table 2**.

**Table 2:** List of gene symbols of MEMRGs.

MEMRGs						
C1QBP	TP53	BDNF	MT-ND4	RYR2	OLA1	CHP1
GFM2	TLR2	NR1H4	ESR1	YWHAZ	RYR3	CHP2
TIGAR	CNR1	CRAT	PSEN1	HTR3A	SSBP1	DAXX
HTT	SCO2	ECHS1	ATP2A2	IDH3A	SURF1	DERL1
CS	UCP1	NDUFS1	CAT	NDUFS3	FLVCR2	GPX1
NFATC4	UCP3	NDUFS2	FOXO1	PDX	IMMT	GRIA1
TANGO2	AK3	NGLY1	NOTCH3	SIRT5	INF2	GRIA2
LIAS	TCF19	DHTKD1	ADK	UQCRC2	NDUFA5	GRIN1
COX5A	DNAJC19	CHCHD10	APOE	XBP1	SMPD2	GRIN2A
ETFA	SUCLA2	GPBAR1	CBS	BSG	ATP5PF	GRIN2B
PPARGC1A	TUFM	PKHD1	CASP7	CKB	GLRX5	GRIN2C
AK2	GFM1	UCHL1	DLD	FUS	MAVS	GRIN2D
CYCS	AUH	GSK3B	EIF2AK3	ID2	MRS2	MAP2K3
FBXL4	HIBCH	ASS1	GLS	IGF2BP2	NDUFAF3	MAP2K6
SLC25A20	TSFM	PRK	HSPD1	SHMT1	NDUFS5	MAP3K5
COQ9	TAFAZZIN	MFN2	PGK1	ATG7	SIRT4	MAPK11
SDHB	MTIF2	ATP7B	SLC25A4	BAD	ACAD10	MAPK12
OGDH	TMEM70	IRS1	STIM1	CLOCK	ALYREF	MAPK13
SLC25A3	OPA3	PAH	YWHAE	DBT	BOLA3	MAPK14
ETFB	SERAC1	SCN9A	HPRT1	EIF2S1	COX7A2L	NEFH
PTEN	CENPO	SOD2	PKD2	ESRRG	FAHD1	NEFL
PDP1	MTIF3	TPI1	SDHA	GDAP1	SLC25A27	NEFM
UCP2	LIX1	KCNK9	ADORA2B	PNPLA6	TIMMDC1	NOS1
LDHA	C2orf88	ACADVL	HSPA9	SLC25A5	ATP5MC1	PPP3CA
EIF4E	NDUFV1	BTD	HTR2A	TFAM	OCIAD1	PPP3CB
RPS6KB1	NDUFS4	GCDH	IL1B	UQCRC1	MT-ATP6	PPP3CC
PRKCZ	NFU1	OCRL	INS	CPT1C	MT-CO1	PPP3R1
KNG1	LIPT2	VDAC1	KCNJ5	EPO	MT-ND1	PPP3R2
POLG	FH	ATP5F1B	RYR1	HTR2B	ALS2	PRPH
DLAT	LIPT1	KRIT1	VCL	LRPPRC	APAF1	PRPH2
EIF4EBP1	IBA57	RARS2	ACOX1	NME4	BAX	RAB5A
ACAD9	SRC	SUMF1	ADORA2A	PDK4	BCL2	RAC1
KLK4	SIRT3	MIPEP	COX4I1	PROK2	BCL2L1	SLC1A2
CR1	ESRRA	SLC27A1	FXN	PTPA	BID	TNF
TNNT1	TPK1	SZT2	HK2	SOAT2	CASP1	TNFRSF1A
CD200	AGK	PPARGC1B	HUWE1	BICD2	CASP3	TNFRSF1B
OMA1	MFF	MITD1	NEU1	DMRT1	CASP9	TOMM40
SOD1	AIFM1	FAM210B	PRDX6	NDUFAF1	CCS	TOMM40L

### 2.2 Differential expression analysis

To increase statistical power, we merged datasets GSE98793 and GSE76826 using the R package *sva* (21) and performed batch effect removal to obtain a unified MDD dataset. Differential expression analysis was performed to identify DEGs between MDD and control samples. We then identified the intersection between these DEGs and the MEMRGs list. Genes exhibiting |logFC| > 0 and a p-value < 0.05 were defined as MEMRDEGs. The intersection was visualized using a Venn diagram. The differential analysis results were further visualized using a volcano plot and a differential ranking plot generated via the R package *ggplot2*.

### 2.3 Machine learning-based gene prioritization and model construction

Least absolute shrinkage and selection operator (LASSO) regression (22) was performed using the R package *glmnet* (23) with 10-fold cross-validation and a random seed of 2022 to screen MEMRDEGs from the combined MDD dataset. Genes with non-zero coefficients at the optimal lambda value were selected. Subsequently, a support vector machine (SVM) model (24) was constructed based on the LASSO-selected MEMRDEGs, optimized for maximum accuracy and minimum error rate.

The overlapping genes identified by both LASSO and SVM were considered key genes. A logistic regression model was then built using these key genes. To ensure the robustness of the identified biomarkers and avoid overfitting—especially given the absence of an external validation set—we employed a strict internal validation strategy. The model was validated using the Bootstrap method (1,000 resamplings) via the R package *rms*. A nomogram was created to visualize the multifactorial relationships and calculate total scores for predicting MDD probability. Model calibration was assessed with calibration curves (based on 1,000 bootstrap resamples), and clinical utility was evaluated via Decision Curve Analysis (DCA) using the *ggDCA* package (25).

### 2.4 ROC analysis

ROC curves (26) were generated using the R package *pROC* to evaluate the diagnostic potential of key gene expressions in distinguishing MDD patients from healthy controls. The area under the curve (AUC) was calculated for each gene to quantify discriminatory power, with values interpreted as follows: 0.5–0.7 indicating low accuracy, 0.7–0.9 moderate accuracy, and > 0.9 high accuracy.

### 2.5 Immune infiltration analysis

The immune cell landscape of each MDD sample was quantified using two distinct algorithms. We utilized single-sample Gene Set Enrichment Analysis (ssGSEA) via the R ‘*GSVA*’ package (27) to derive enrichment scores for various immune populations. In parallel, the CIBERSORT (28) deconvolution algorithm, referenced against the LM22 signature matrix, was used to estimate the relative fractions of 22 immune cell types. The resulting immune profiles from both methods were then compared between the MDD and control cohorts, and between high- and low-MEMscore subgroups, with differences visualized by boxplots. Pearson correlation was employed to assess the relationships among immune cell types and between immune cells and key genes, with findings visualized using dot plots and network diagrams via the ‘*ggplot2*’ package.

### 2.6 Single-cell eQTL MR analysis

To investigate the causal effects of 11 pre-identified key genes on MDD risk, we performed a cell-type-stratified two-sample MR analysis. Genetic instruments for gene expression in 14 immune cell types were sourced from the Yazar et al. single-cell eQTL (sc-eQTL) catalogue (29). Summary-level data for the MDD outcome were obtained from the Nagel et al. GWAS meta-analysis (N = 357,957 European-ancestry individuals) (30).

For each gene-cell pair, instrumental variables (IVs) were rigorously selected based on three primary criteria to ensure validity and specificity: (i) Cis-regulatory definition: only SNPs located within 1 Mb of the transcription start site of the target gene were considered to capture direct regulatory effects and minimize horizontal pleiotropy; (ii) Significance threshold: SNPs demonstrating a genome-wide significance of *P* < 5 × 10^-5^ were selected; and (iii) Independence: linkage disequilibrium (LD) clumping was performed (r² < 0.1 within a 1000 kb window) to remove correlated variants. To avoid weak instrument bias, the F-statistic was calculated for each gene-cell pair. In the final selection, F-statistics for individual SNPs ranged from 10.10 to 264.65, all exceeding the conventional threshold of 10 (F >10).

Primary causal estimates were calculated using the inverse-variance weighted (IVW) method utilizing the *TwoSampleMR* R package (31). To evaluate robustness, sensitivity analyses were conducted. Heterogeneity among IVs was assessed using Cochran’s Q statistic. Potential directional pleiotropy was evaluated using the MR-Egger intercept test, and horizontal pleiotropy was further examined via the MR-PRESSO global test. Additionally, leave-one-out analyses were performed to verify that the causal associations were not driven by any single influential SNP.

### 2.7 Animal experiments

#### 2.7.1 Animal preparation

Animal experiments were conducted in accordance with the ARRIVE guidelines (32) and relevant institutional regulations, and were approved by the Research Ethics Committee of Fujian University of Traditional Chinese Medicine (approval no. 2019-FUCM-2022104). Given the higher prevalence of depression in females compared to males, female rats were used to model sex-specific biological and behavioral characteristics (33). Twelve 12-week-old healthy female Sprague-Dawley (SD) rats (body weight: 200 ± 10 g) were obtained from Shanghai SLAC Laboratory Animal Co., Ltd. (license no. SCXK (Shanghai) 2022-0004; qualification certificate no. 20220004002603). Rats were housed in the Animal Experiment Center at Fujian University of Traditional Chinese Medicine under controlled conditions: temperature of 22 ± 2 °C, 12-h light/dark cycle, and humidity of 40–60%. Animals were randomly assigned to two groups (n = 6 per group): a control group and a chronic unpredictable mild stress (CUMS) group. All rats had ad libitum access to food and water, except during experimental procedures.

#### 2.7.2 CUMS modeling

The CUMS model is widely regarded as a classic paradigm for simulating the core pathological features of MDD (34). The CUMS model was established over 21 days to induce depressive-like behaviors in rats. One stressor was randomly selected each day, ensuring no repetition on consecutive days. Stressors included: 24-h food deprivation, 24-h water deprivation, 24-h cage tilting (45°from horizontal), 24-h wet bedding (200 mL water per 100 g sawdust), 24-h restraint, 5-min tail pinching (1 cm from the tail base), 10-min ice-water swimming (4 °C), and 15-min heat stress (45 °C). Rats were randomly assigned to control or CUMS groups (n = 6 per group) as described in Section 2.7.1, with ad libitum access to food and water except during stressors.

#### 2.7.3 Sucrose preference test and open field test

The sucrose preference test (SPT) was performed to evaluate anhedonia in rats. On days 1–2, two bottles of 1% (w/v) sucrose solution were provided in each cage for habituation. Following 12-h food and water deprivation, one bottle was replaced with water. After 1 h, bottle positions were swapped to minimize positional bias. Consumption was measured after an additional 2 h, and sucrose preference was calculated as: (sucrose intake/total fluid intake) × 100%.

The open field test (OFT) was conducted to assess anxiety-like behaviors. In a quiet environment, each rat was placed in the center of an open field arena. Locomotor activity, including total distance traveled, was recorded for 5 min using TopScanLite software (version 2.00). The arena was cleaned with 75% ethanol between trials to eliminate olfactory cues.

#### 2.7.4 Hematoxylin-eosin staining

Hippocampal tissues were fixed, paraffin-embedded, and sectioned coronally (6 μm thickness). Sections were baked at 60 °C for 2 h, deparaffinized in xylene (two changes, 10 min each), and rehydrated through a graded ethanol series (100% twice, 95%, 90%, 80%; 3 min each) followed by distilled water. Sections were stained with hematoxylin for 4 min, rinsed in tap water (three times), differentiated in 1% acid alcohol for 2 s, and counterstained with eosin for 50 s. Dehydration was performed through graded ethanol (80%, 90%, 95%, 100% twice; 2 s each), followed by clearing in xylene (two changes, 2 min each). Slides were mounted with neutral resin and observed under a light microscope at 200× magnification to assess morphological changes in the hippocampal tissue.

#### 2.7.5 Nissl staining

Paraffin sections (6 μm) were deparaffinized and rehydrated as described in Section 2.7.4. Sections were stained with cresyl violet (Yuanye Bio-Technology, Shanghai, China) at 56 °C for 1 h, differentiated in 0.2% acetic acid until optimal contrast was achieved, and rapidly dehydrated in absolute ethanol (two changes). Slides were cleared in xylene (two changes) and mounted with neutral resin. Neuronal morphology in the hippocampus was examined under a light microscope at 200× magnification.

#### 2.7.6 Reverse transcription quantitative polymerase chain reaction analysis

Total RNA was extracted from hippocampal tissues using TRIzol reagent (Invitrogen, Carlsbad, CA, USA), and RNA purity and concentration were assessed via spectrophotometry. Complementary DNA (cDNA) was synthesized using the Premix Ex Taq™ II kit (Takara Biotechnology Co., Ltd., Dalian, China). RT-qPCR was performed on a QuantStudio™ 6 Flex Real-Time PCR System (Applied Biosystems, Foster City, CA, USA) with the same kit, using *β*-actin as the internal reference gene. Relative mRNA expression levels were calculated using the 2^-ΔΔCt^ method. Results are presented in **Table 3**.

**Table 3:** Primer sequences used in the RT-qPCR.

Gene	Primer sequences
*SOD1*	Forward: 5′-GCGTCATTCACTTCGAGCAG -3′
	Reverse: 5′-ACATGCCTCTCTTCATCCGC -3′
*UCP2*	Forward:5′-AGCAGTTCTACACCAAGGGC-3′
	Reverse: 5′-GCTCTGGTATCTCCGACCAC-3′
*β*-actin	Forward:5′-GGAGATTACTGCCCTGGCTCCTA-3′
	Reverse: 5′-GACTCATCGTACTCCTGCTTGCTG-3′

#### 2.7.7 Protein detection

*SOD1*, *UCP2*, and GAPDH (internal reference) were quantified using an automated capillary western blot system (ProteinSimple, San Jose, CA, USA). Hippocampal tissues were homogenized in RIPA lysis buffer supplemented with protease inhibitors, and total protein concentration was determined using a BCA assay kit (Beyotime Biotechnology, Shanghai, China). Samples were diluted to a standard concentration, mixed with 5× Fluorescent Master Mix (containing DTT and SDS) at a 1:4 ratio, and denatured at 95 °C for 5 min. The assay plate was loaded according to the manufacturer’s instructions: 3 μL of protein sample, 10 μL of antibody diluent, and 10 μL of primary antibodies were dispensed into respective wells. The primary antibodies used were: anti-*SOD1* (1:50 dilution; Cat<ns/> YM8065, Immunoway), anti-*UCP2* (1:50 dilution; Cat<ns/> YM9201, Immunoway), and anti-GAPDH (1:2200 dilution; Cat<ns/> 60004-1-Ig, Proteintech). Secondary antibody (anti-rabbit, 10 μL), luminol-peroxide solution (15 μL), and wash buffer (500 μL) were added to designated wells. Plates were centrifuged at 2500 rpm for 5 min at room temperature (25 °C) and analyzed on the Jess automated system (ProteinSimple). Band intensities were quantified using Compass software (version 4.0.0), with relative protein expression calculated as the ratio of target band intensity to GAPDH.

### 2.8 Statistical analysis

Data were analyzed using R software (version 4.2.3) and SPSS (version 26.0). Continuous variables were expressed as mean ± standard deviation (SD). Comparisons between two groups were performed using the independent Student’s t-test for normally distributed data or the Wilcoxon rank-sum test for non-normal data. For comparisons among three or more groups, the Kruskal-Wallis test was applied. Categorical variables were analyzed using the chi-square test or Fisher’s exact test, as appropriate. ROC curves were generated using the *pROC* package in R. Correlations were assessed via Spearman’s rank correlation coefficient. For Single-cell eQTL Mendelian randomization (Section 2.6), causal estimates were derived using the *TwoSampleMR* package in R, with sensitivity analyses as described. All tests were two-tailed, with statistical significance set at *P* < 0.05. In figures, significance levels are indicated as follows: ns (not significant, *P* ≥ 0.05); * (*P* < 0.05); ** (*P* < 0.01); *** (*P* < 0.001).

## 3 Results

### 3.1 Analysis flow chart

**Figure 1:**
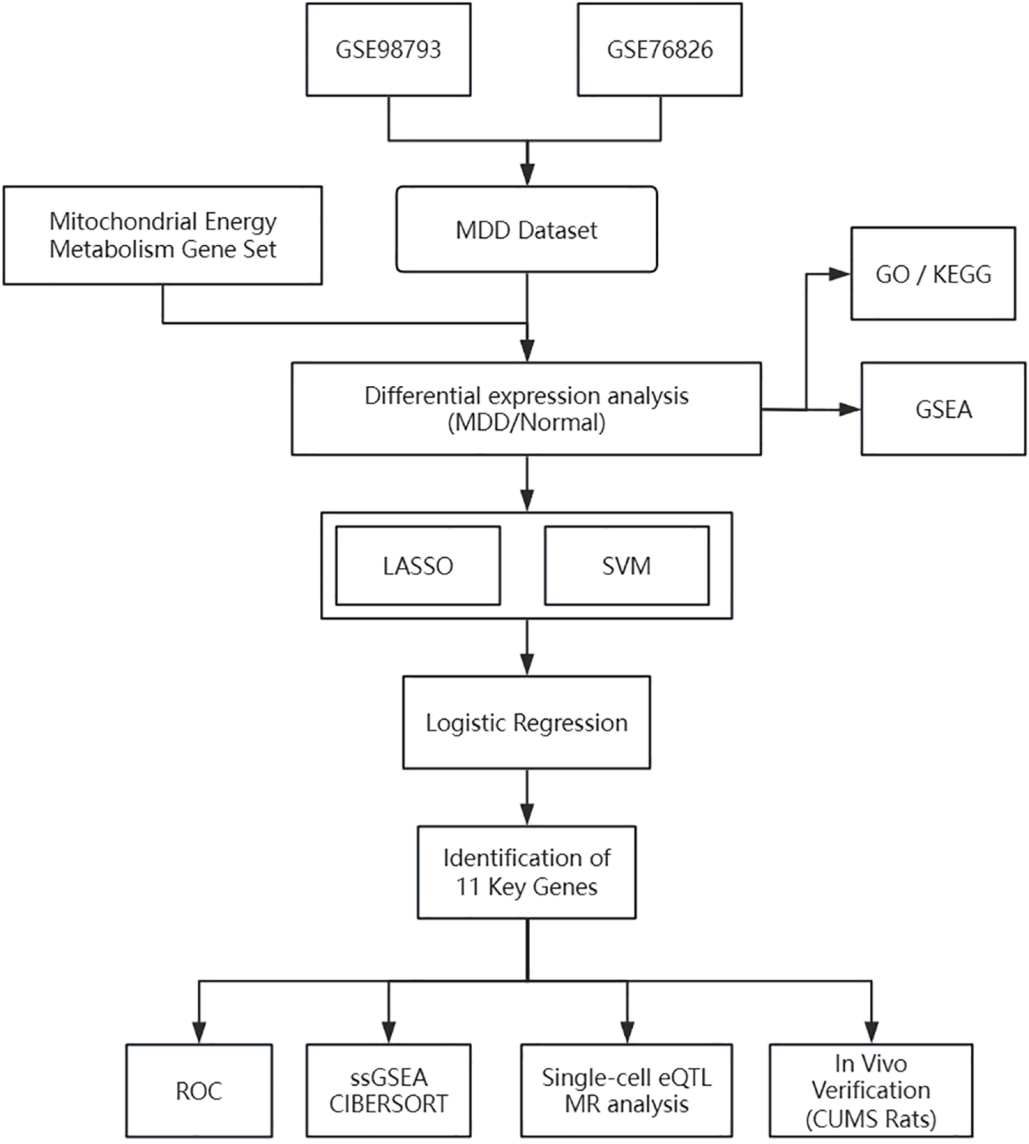
Technology roadmap.

The technical approach for this bioinformatics analysis is illustrated below (**Figure 1**).

### 3.2 Differential expression analysis

**Figure 2:**
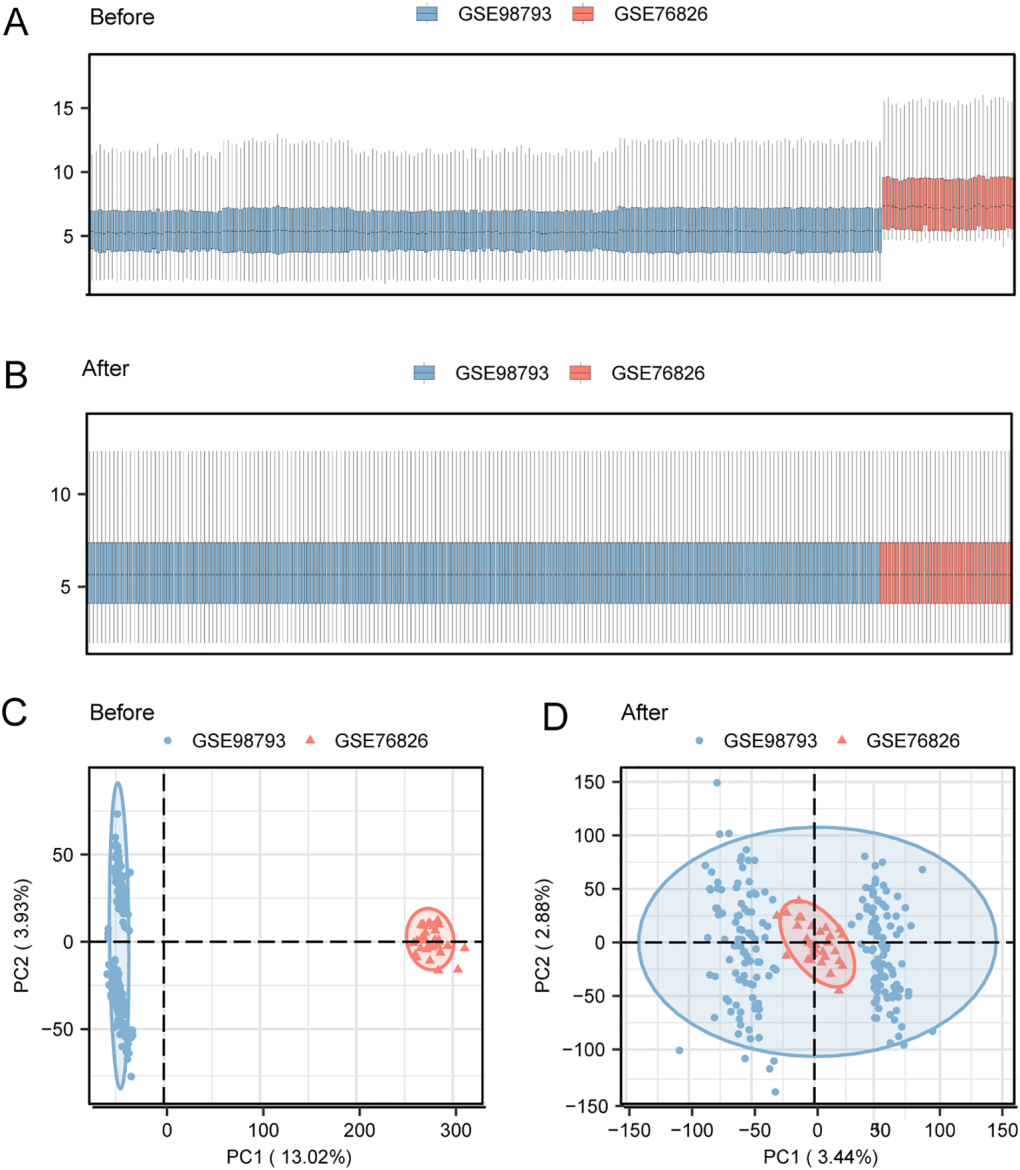
Batch effect correction in the merged MDD dataset. **(A)** Boxplot of gene expression distributions in the merged MDD dataset before batch effect correction. **(B)** Boxplot of gene expression distributions in the merged MDD dataset after batch effect correction. **(C)** Principal component analysis (PCA) plot of the MDD dataset before correction. **(D)** PCA plot of the MDD dataset after correction.

Batch effects in datasets GSE98793 and GSE76826 were removed using the R package *sva*, resulting in a merged GEO dataset designated as the MDD dataset. The efficacy of batch effect removal was confirmed by comparing distribution boxplots and principal component analysis (PCA) plots before and after adjustment (**Figures 2A–D**), which demonstrated effective homogenization of sample distributions.

**Figure 3:**
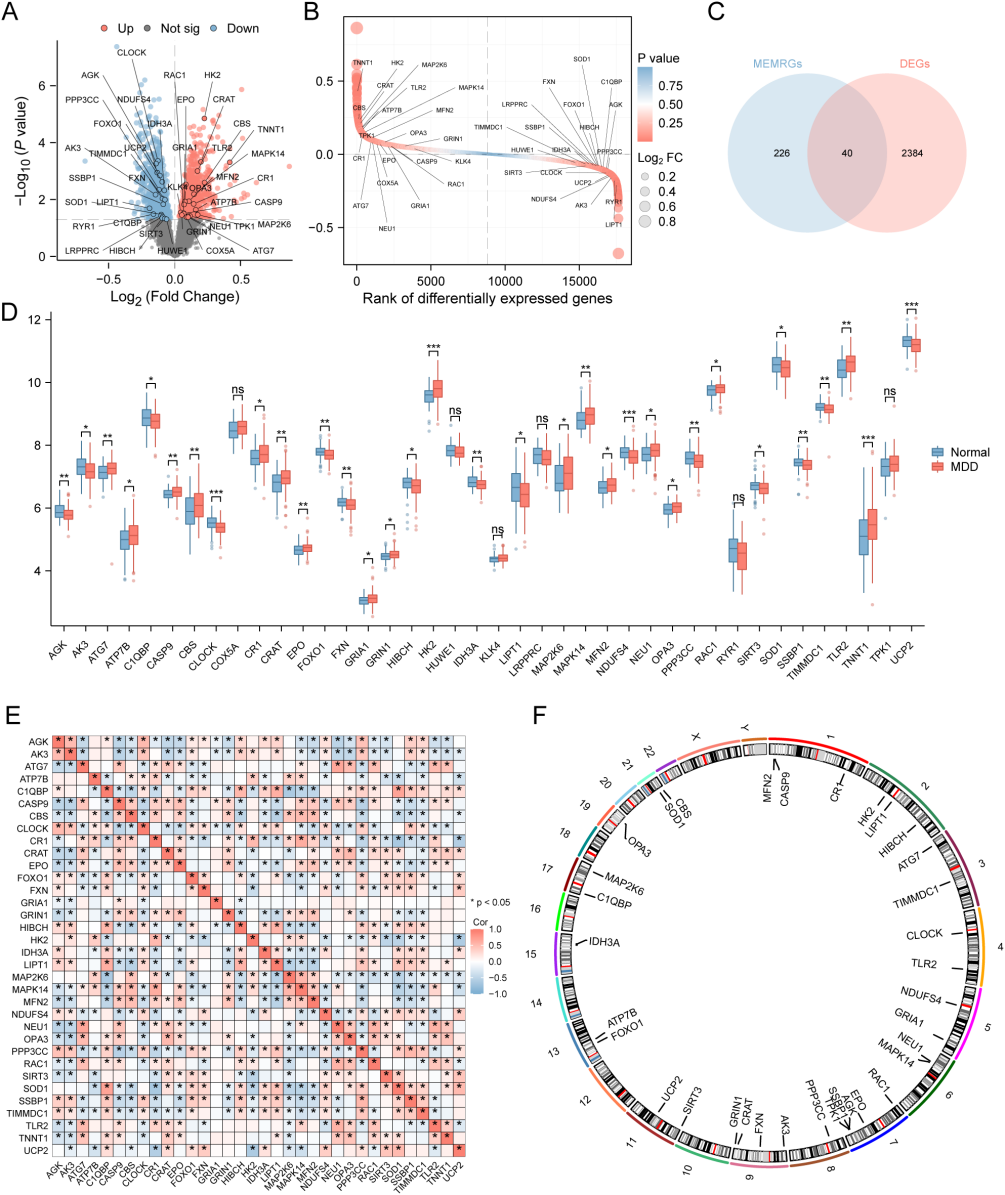
Differential expression analysis in the merged MDD dataset. **(A)** Volcano plot of differentially expressed genes (DEGs) between the normal and MDD groups. **(B)** Ranking plot of DEGs, with the x-axis representing the position after sorting by log2 fold change (log2FC) and the y-axis representing log2FC values. **(C)** Venn diagram illustrating the overlap between DEGs and MEMRGs in the merged dataset. **(D)** Boxplot comparing expression levels of MEMRDEGs between groups. **(E)** Correlation heatmap of MEMRDEGs. **(F)** Chromosomal localization of MEMRDEGs. Significance levels are indicated by asterisks (see Methods for details).

Differential expression analysis of the MDD dataset identified 2424 differentially expressed genes (DEGs) based on |log2FC| > 0 and *P* < 0.05 criteria, including 1115 upregulated and 1309 downregulated genes in the MDD group relative to controls. Results were visualized using a volcano plot and a ranking plot generated with the R package *ggplot2* (**Figures 3A, B**). Intersection of DEGs with MEMRGs yielded 40 MEMRDEGs: *AGK, AK3, ATG7, ATP7B, C1QBP, CASP9, CBS, CLOCK, COX5A, CR1, CRAT, EPO, FOXO1, FXN, GRIA1, GRIN1, HIBCH, HK2, HUWE1, IDH3A, KLK4, LIPT1, LRPPRC, MAP2K6, MAPK14, MFN2, NDUFS4, NEU1, OPA3, PPP3CC, RAC1, RYR1, SIRT3, SOD1, SSBP1, TIMMDC1, TLR2, TNNT1, TPK1*, and *UCP2* (**Figure 3C**).

To ensure robustness, we re-verified the differential expression status of these 40 candidates within our final merged dataset. This confirmation step revealed that 34 of the genes robustly met the significance threshold (*P* < 0.05), while six (*COX5A*, *HUWE1*, *KLK4*, *LRPPRC*, *TPK1*, and *RYR1*) did not (**Figure 3D**). Consequently, these 34 confirmed MEMRDEGs were selected for all subsequent analyses. Correlations among the 34 MEMRDEGs were illustrated in a heatmap (**Figure 3E**), revealing an equal number of positive and negative correlations. Chromosomal locations of the MEMRDEGs were annotated using the *RCircos* package (**Figure 3F**), with five genes localized to chromosome 7 (*AGK*, *EPO*, and others as shown).

### 3.3 LASSO, SVM, logistics screening genes

**Figure 4:**
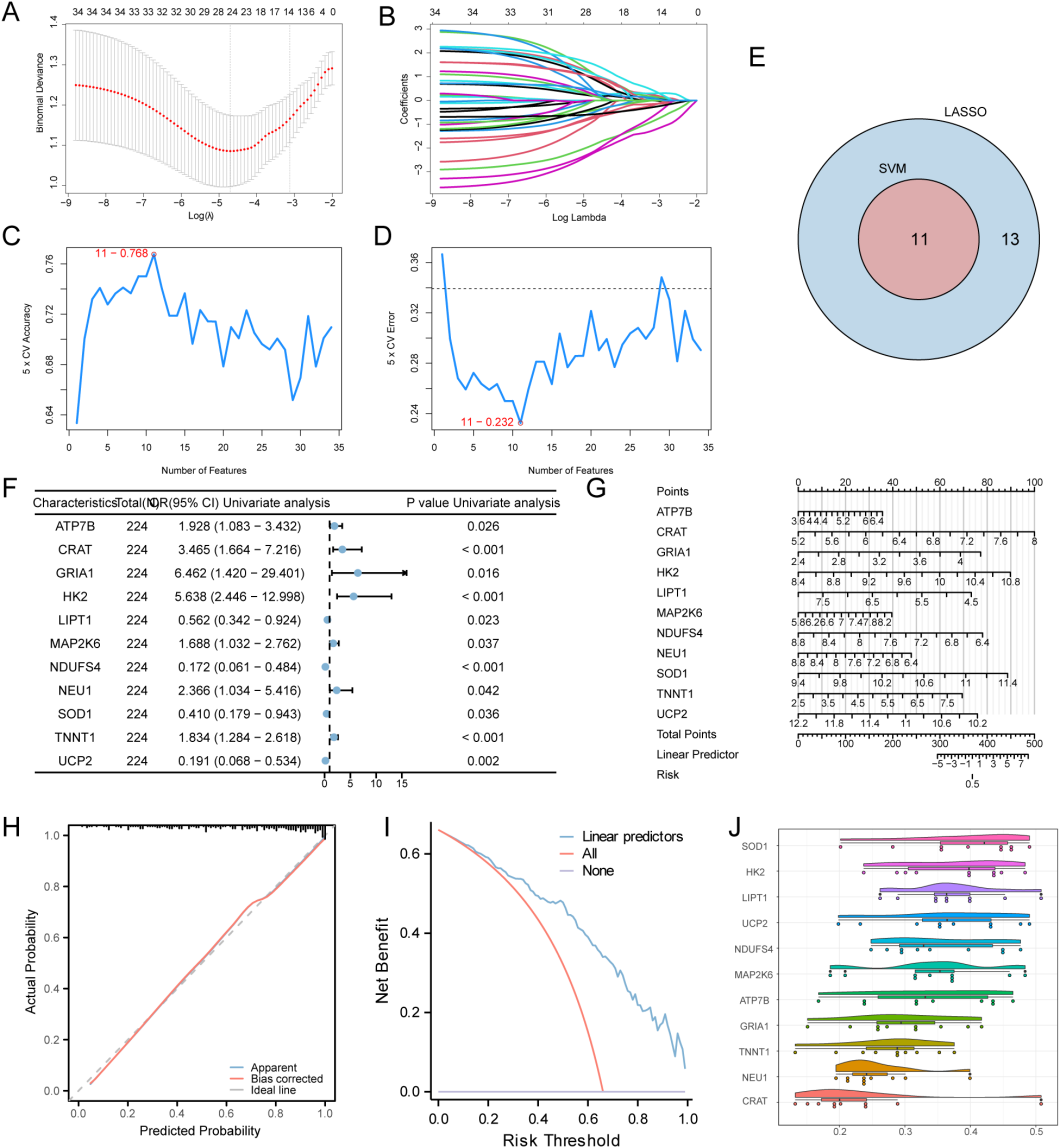
Construction of the MEMRDEGs diagnostic model. **(A)** LASSO regression model for MEMRDEGs. **(B)** Variable trajectory plot from the LASSO regression model. **(C)** Number of genes yielding the highest accuracy via the SVM algorithm. **(D)** Number of genes yielding the lowest error rate via the SVM algorithm. **(E)** Venn diagram showing the intersection of genes screened by LASSO regression and SVM algorithms. **(F)** Forest plot of logistic regression analysis results. **(G)** Nomogram derived from the logistic regression model. **(H)** Calibration curve for the logistic predictive model, assessing prediction accuracy. **(I)** DCA plot, with threshold probability on the x-axis and net benefit on the y-axis. **(J)** Functional similarity analysis of key genes.

To ascertain the diagnostic significance of MEMRDEGs within the MDD dataset, LASSO regression analysis was utilized to construct a diagnostic model (**Figure 4A**). The LASSO regression results, including the variable trajectory diagram, are visualized (**Figure 4B**). This analysis identified an optimal signature consisting of 11 MEMRDEGs, which yielded the highest diagnostic accuracy (**Figure 4C**) and the lowest cross-validation error rate (**Figure 4D**). The results indicate that the SVM model achieves maximum accuracy with 11 genes: *ATP7B, CRAT, GRIA1, HK2, LIPT1, MAP2K6, NDUFS4, NEU1, SOD1, TNNT1*, and *UCP2*. The genes identified by both algorithms were intersected, as demonstrated in a Venn diagram (**Figure 4E**), yielding 11 intersecting MEMRDEGs: *ATP7B, CRAT, GRIA1, HK2, LIPT1, MAP2K6, NDUFS4, NEU1, SOD1, TNNT1*, and *UCP2*. Logistic regression analysis was conducted on these 11 MEMRDEGs to build a logistic regression model. Through this process, the 11 MEMRDEGs were confirmed as key genes (**Table 4**). The findings from the univariate logistic regression analysis were organized and presented in a forest plot (**Figures 4F, G**).

**Table 4:** Description of key genes.

ID	Description	logFC	AveExpr	t	p-value	B
ATP7B	ATPase Copper Transporting Beta	0.157414	5.080752	2.290433	0.022917	-3.50121
CRAT	Carnitine O-Acetyltransferase	0.196384	6.896433	3.546039	0.000475	-0.21955
GRIA1	Glutamate Ionotropic Receptor AMPA Type Subunit 1	0.069075	3.10261	2.452934	0.014926	-3.15083
HK2	Hexokinase 2	0.222127	9.704779	4.438575	1.41E-05	2.869647
LIPT1	Lipoyltransferase 1	-0.19147	6.465154	-2.33666	0.02033	-3.40384
MAP2K6	Mitogen-Activated Protein Kinase Kinase 6	0.171996	7.041615	2.128196	0.034402	-3.82819
NDUFS4	NADH: Ubiquinone Oxidoreductase Subunit S4	-0.14208	7.681223	-3.50741	0.000546	-0.33963
NEU1	Neuraminidase 1	0.098445	7.776098	2.079012	0.038743	-3.92278
SOD1	Superoxide Dismutase 1	-0.10316	10.47319	-2.13788	0.033599	-3.80932
TNNT1	Troponin T1, Slow Skeletal Type	0.413491	5.361961	3.537982	0.000489	-0.24469
UCP2	Uncoupling Protein 2	-0.13363	11.22717	-3.29874	0.001128	-0.96785

Calibration analysis was performed to assess the accuracy and resolution of the diagnostic model, resulting in a calibration curve (**Figure 4H**). The curve illustrates the alignment between the optimal theoretical probability (solid line) and the model-predicted probability (dashed line), evaluating the model’s predictive accuracy for actual outcomes. DCA was implemented to validate the model’s clinical utility, with results presented (**Figure 4I**). The DCA indicates that the model provides a higher net benefit than ‘All positive’ and ‘All negative’ strategies within a specific range, demonstrating superior performance. As shown (**Figures 4H, I**), the constructed model exhibits high accuracy in diagnosing MDD. Functional similarity analysis was conducted on the 11 key genes to elucidate their relationships, with results visualized in a cloud and rain map (**Figure 4J**). The analysis reveals that *SOD1* exhibits the highest functional similarity value compared to the other key genes.

### 3.4 Diagnostic ROC analysis

**Figure 5:**
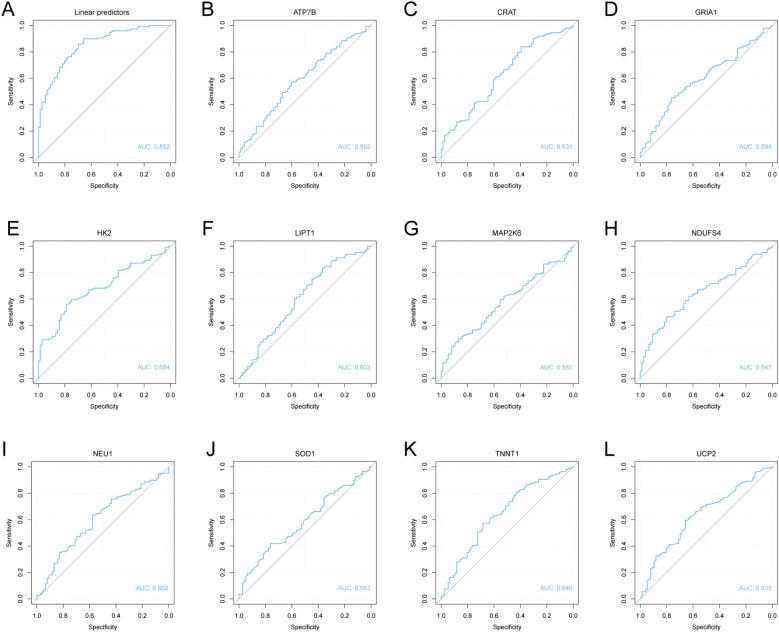
ROC curve analysis of the diagnostic model and key genes in the MDD dataset. **(A)**. ROC curve of the logistic regression model linear predictors. **(B–L)**. ROC curves for the 11 key genes (ATP7B, CRAT, GRIA1, HK2, LIPT1, MAP2K6, NDUFS4, NEU1, SOD1, TNNT1, and UCP2), with MDD and normal groups as outcome variables. The area under the curve (AUC) ranges generally between 0.5 and 1; values closer to 1 indicate better diagnostic performance. AUC values of 0.5–0.7 suggest low accuracy, 0.7–0.9 indicate moderate accuracy, and >0.9 reflect high accuracy.

ROC curves were constructed using linear predictors from the logistic regression model to evaluate its diagnostic performance in the MDD dataset. The combined signature demonstrated good diagnostic accuracy in distinguishing MDD cases from controls, achieving AUC of 0.852 (**Figure 5A**). In contrast, the diagnostic utility of individual genes was limited, with AUCs ranging from 0.581 to 0.684 (**Figures 5B–L**).

### 3.5 ssGSEA immune infiltration analysis between MDD and normal groups in the MDD dataset

**Figure 6:**
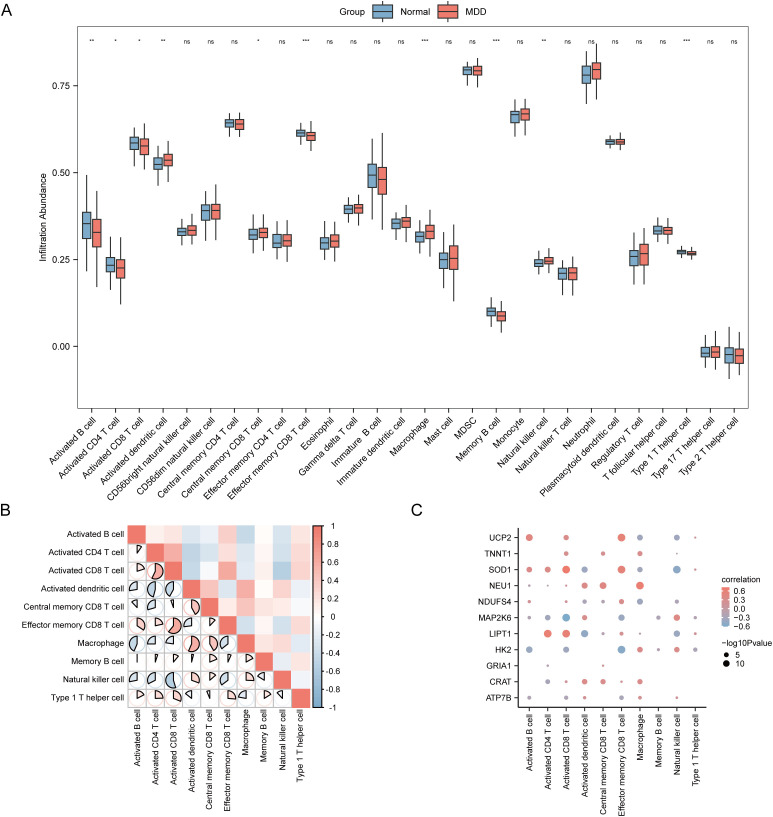
ssGSEA immune infiltration analysis in the MDD dataset. **(A)** Boxplot comparing ssGSEA immune infiltration scores between MDD and normal groupsin the MDD dataset. **(B)** Correlation heatmap of immune cell infiltration abundance in the MDD dataset. **(C)** Correlation plot between immune cellsand key genes in the MDD dataset. ns, not significant (P ≥ 0.05); *P < 0.05; **P < 0.01; ***P < 0.001.

Immune cell infiltration was evaluated using the ssGSEA algorithm to examine differences between MDD and normal controls in the MDD dataset. Infiltration levels of 28 immune cell types were assessed, and the Mann-Whitney U test was applied to compare these levels between groups. The results are presented in a comparative chart (**Figure 6A**). Statistically significant differences (*P* < 0.05) were observed in the infiltration levels of ten immune cell types: activated CD4 T cells, central memory CD8 T cells, activated CD8 T cells, activated B cells, effector memory CD8 T cells, memory B cells, type 1 T helper cells, activated dendritic cells, macrophages, and natural killer cells. Correlation analysis among these 10 dysregulated cell types revealed a strong positive association between effector memory and activated CD8+ T cells and a strong negative association between activated CD8+ T cells and natural killer cells (**Figure 6B**).

Next, we investigated the relationship between the 11 key genes and these 10 immune cell populations (**Figure 6C**). Several strong positive correlations emerged. Specifically, *HK2* expression correlated with macrophages and natural killer cells. *SOD1* and *UCP2* expression were associated with multiple activated adaptive immune cells, including activated CD4+/CD8+ T cells and B cells. Furthermore, *NEU1* expression correlated positively with macrophages and activated dendritic cells, while *NDUFS4* was associated with effector memory CD8+ T cells (all *P* < 0.05). These correlations suggest that MEMRGs may modulate immune infiltration patterns in MDD, laying the groundwork for causal investigations.

### 3.6 CIBERSORT immune infiltration analysis between MDD and normal groups in the MDD dataset

**Figure 7:**
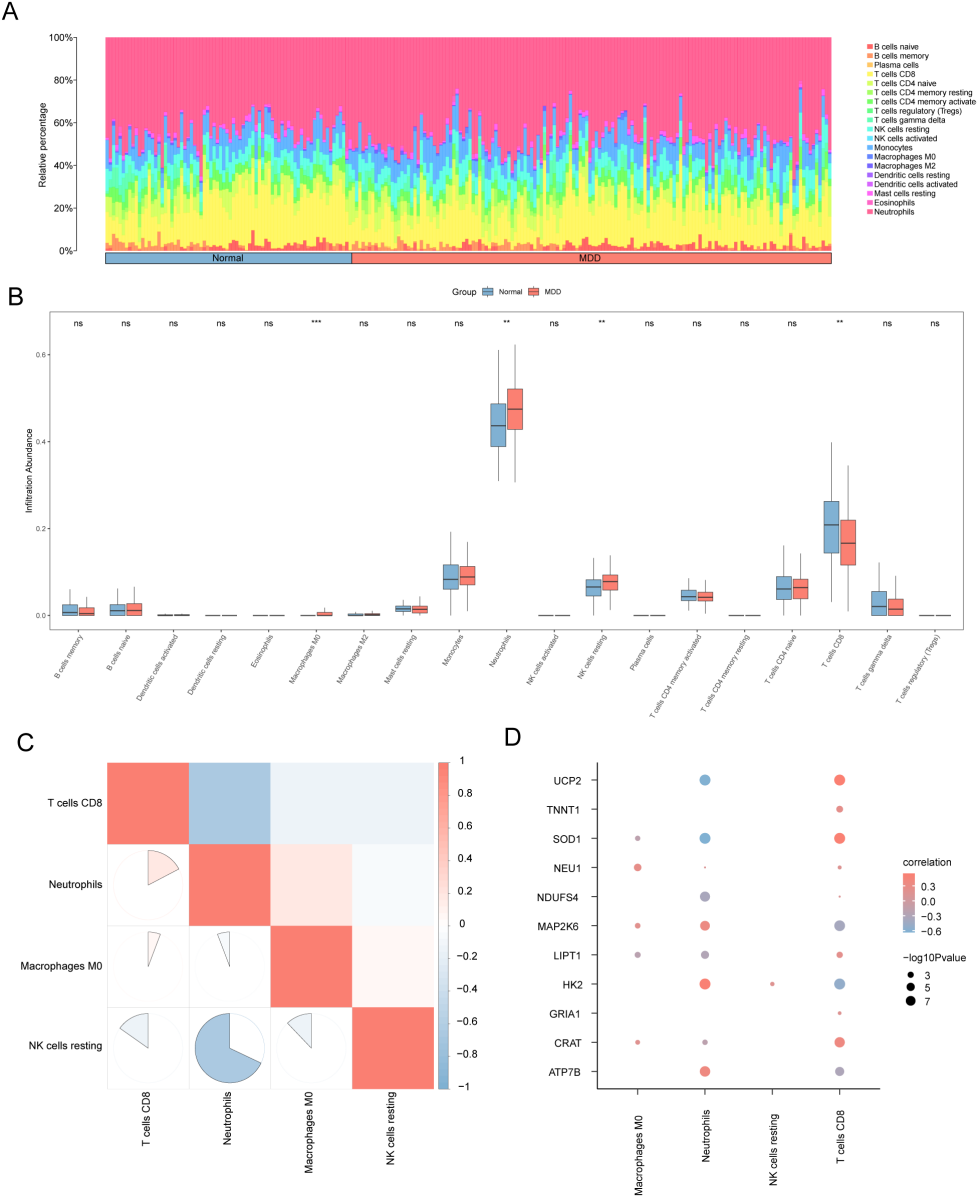
CIBERSORT immune cell infiltration analysis in the MDD dataset. **(A)** Stacked histogram showing the relative proportions of immune cell infiltration inMDD and normal groups. **(B)** Group comparison chart of immune cell infiltration levels between MDD and normal groups. **(C)** Correlation analysis ofimmune cell infiltration abundance. **(D)** Correlation diagram between immune cell infiltration levels and expression of key genes. ns, not significant (P ≥ 0.05); **P < 0.01; ***P < 0.001.

To complement the ssGSEA findings and validate the altered immune landscape using an alternative deconvolution algorithm, we next employed CIBERSORT. This analysis confirmed significant heterogeneity in immune profiles (**Figure 7A**) and revealed that MDD patients had significantly higher proportions of four immune cell types: M0 macrophages (*P* < 0.001), neutrophils, resting NK cells, and CD8+ T cells (all *P* < 0.01) (**Figure 7B**). Among these dysregulated cells, M0 macrophages and resting NK cells were strongly positively correlated, while neutrophils showed a strong negative correlation with resting NK cells (**Figure 7C**).

We then correlated the proportions of these four cell types with the expression of our 11 key genes (**Figure 7D**). Both *SOD1* and *UCP2* expression were positively associated with CD8+ T cell abundance. *NEU1* and *HK2* expression also showed positive correlations with M0 macrophage levels. These findings align with ssGSEA results, reinforcing the link between MEMRG expression and altered immune profiles in MDD, and highlighting potential pathways for immune-mediated disease mechanisms.

### 3.7 Causal effects of gene expression on MDD risk assessed by MR

The preceding analyses revealed significant correlations between the expression of 11 key genes and the infiltration levels of various immune cells (Sections 3.5 & 3.6). To investigate whether these associations reflect a causal relationship between gene expression within specific immune cell types and MDD pathogenesis, we conducted a two-sample MR analysis.

**Figure 8:**
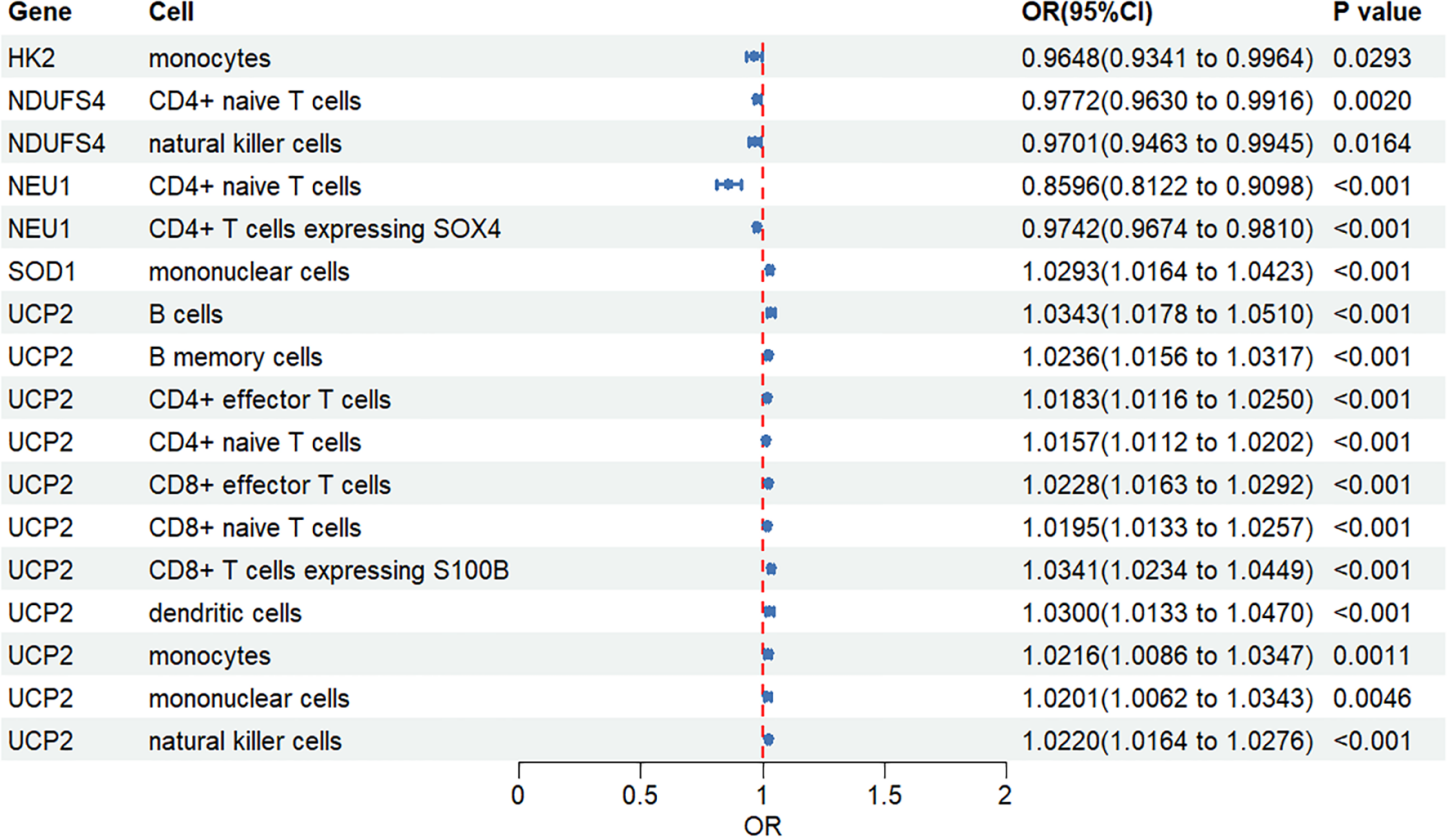
Mendelian randomization analysis of the causal effects of gene expression on MDD risk. The forest plot illustrates the causal estimates from the two-sample MR analysis. The analysis assessed the effect of the expression of five key genes (HK2, NDUFS4, NEU1, SOD1, and UCP2) in various immune cell types on the risk of MDD. Each point estimate represents the Odds Ratio (OR), and the horizontal lines indicate the 95% Confidence Intervals (CI). The vertical dashed line at OR = 1 represents the null effect. ORs, 95% CIs, and corresponding P-values are listed on the right.

Our MR analysis provided genetic evidence for a causal link between the expression of five key genes in distinct immune populations and the risk of MDD (**Figure 8**).

Protective Causal Effects: We identified several genes whose elevated expression was causally associated with a reduced risk of MDD. Specifically, increased expression of *HK2* in monocytes (OR = 0.96, 95% CI: 0.93-1.00, *P* = 0.029), *NDUFS4* in both CD4+ naive T cells (OR = 0.98, 95% CI: 0.96-0.99, *P* = 0.002) and natural killer cells (OR = 0.97, 95% CI: 0.95-0.99, *P* = 0.016), and *NEU1* in CD4+ naive T cells (OR = 0.86, 95% CI: 0.81-0.91, *P* < 0.001) were identified as protective factors against MDD.

Risk-Conferring Causal Effects: Conversely, the analysis revealed that elevated expression of *SOD1* in mononuclear cells was causally linked to an increased risk of MDD (OR = 1.03, 95% CI: 1.02-1.04, *P* < 0.001). More strikingly, increased expression of *UCP2* was consistently associated with a higher risk of MDD across a broad spectrum of immune cells. These included B cells (OR = 1.03, 95% CI: 1.02-1.05, *P* < 0.001), multiple T cell subsets, monocytes, dendritic cells, and natural killer cells (all *P* < 0.01). The strongest risk effects for *UCP2* were observed in B cells and S100B-expressing CD8+ T cells (OR = 1.03, 95% CI: 1.02-1.04, *P* < 0.001).

The validity of these causal estimates was confirmed through a battery of sensitivity analyses. We found no evidence of significant heterogeneity (Cochran’s Q test, all *P* > 0.05) or directional pleiotropy, as indicated by non-significant MR-Egger regression intercepts (*P* > 0.05) and visually symmetrical funnel plots. Additionally, MR-PRESSO analyses did not detect any outlier genetic variants, and leave-one-out analyses demonstrated that the results were not driven by any single instrumental variable. Detailed results of all sensitivity analyses are provided in the **Supplementary Materials**. Collectively, these rigorous checks affirm the robustness of our findings.

Collectively, these MR findings provide robust genetic evidence supporting a causal role for altered gene expression within specific immune cell populations in the etiology of MDD, highlighting both protective and risk-conferring molecular pathways that warrant further investigation.

### 3.8 *In vivo* validation and characterization of key gene expression in a CUMS model

**Figure 9:**
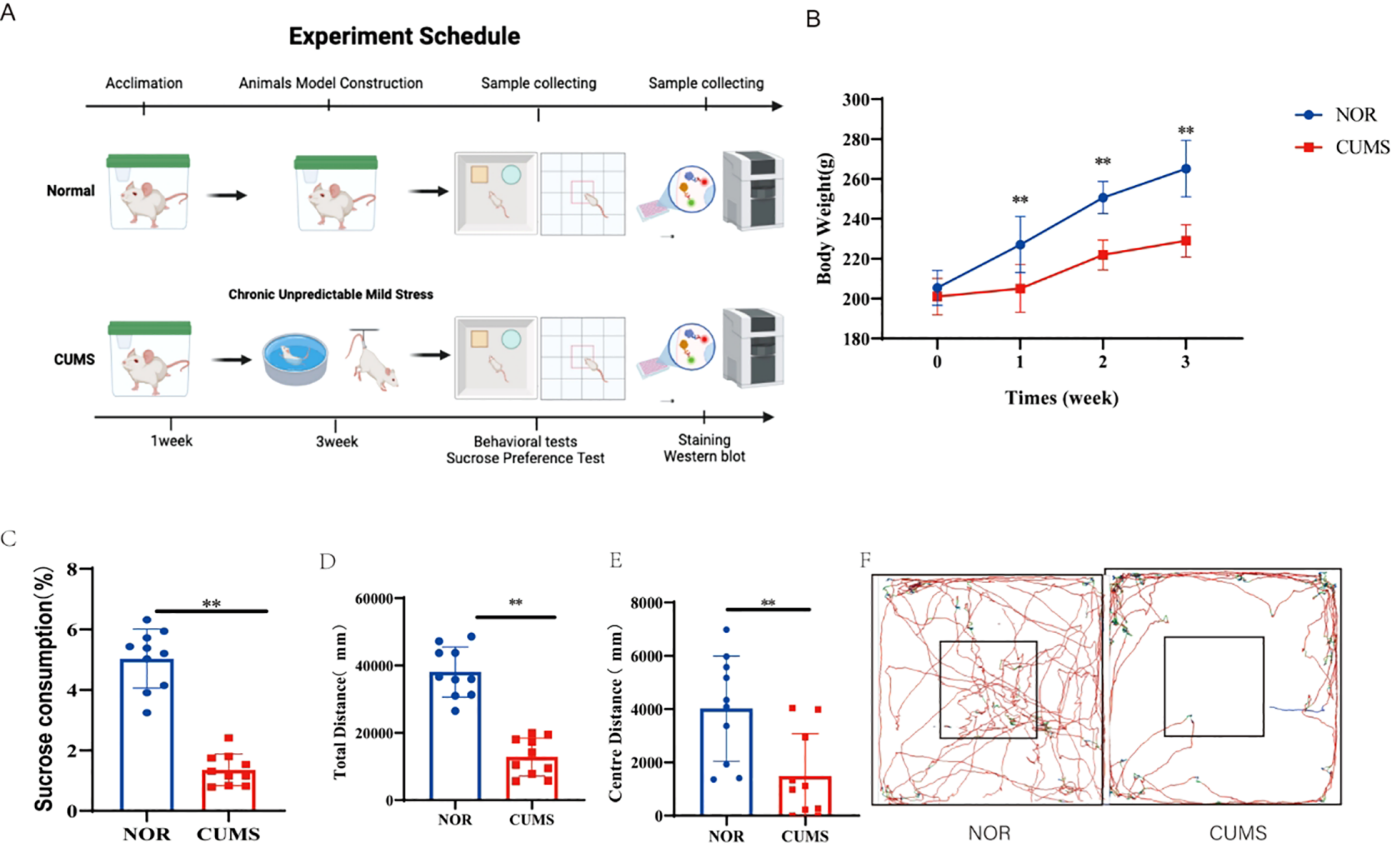
Behavioral validation of the CUMS model in rats. **(A)** Timeline of the experimental design. **(B)** Body weight monitoring indicated suppressed weight gain in CUMS rats. **(C)** Sucrose preference test results showing anhedonia-like behavior. (D–F). Locomotor and anxiety-like behaviors assessed by the open field test. Panels show total distance **(D)**, center zone distance **(E)**, and representative tracking paths **(F)**. Data are presented as mean ± SD (n = 6 per group). **P < 0.01 vs. NOR group.

To investigate the *in vivo* relevance of our primary findings, we established a CUMS rat model to mimic depressive-like behaviors (experimental schematic in **Figure 9A**). The successful induction of a depressive phenotype was first confirmed through behavioral and physiological assessments. Rats subjected to CUMS exhibited significantly lower body weight gain compared to the control group (*P* < 0.05, **Figure 9B**). Furthermore, the CUMS group displayed a marked reduction in sucrose preference, a core indicator of anhedonia (*P* < 0.05, **Figure 9C**). In the open field test, CUMS-exposed rats showed significantly decreased total distance traveled and time spent in the center zone, indicative of locomotor inhibition and anxiety-like behavior (*P* < 0.05, **Figures 9D–F**).

**Figure 10:**
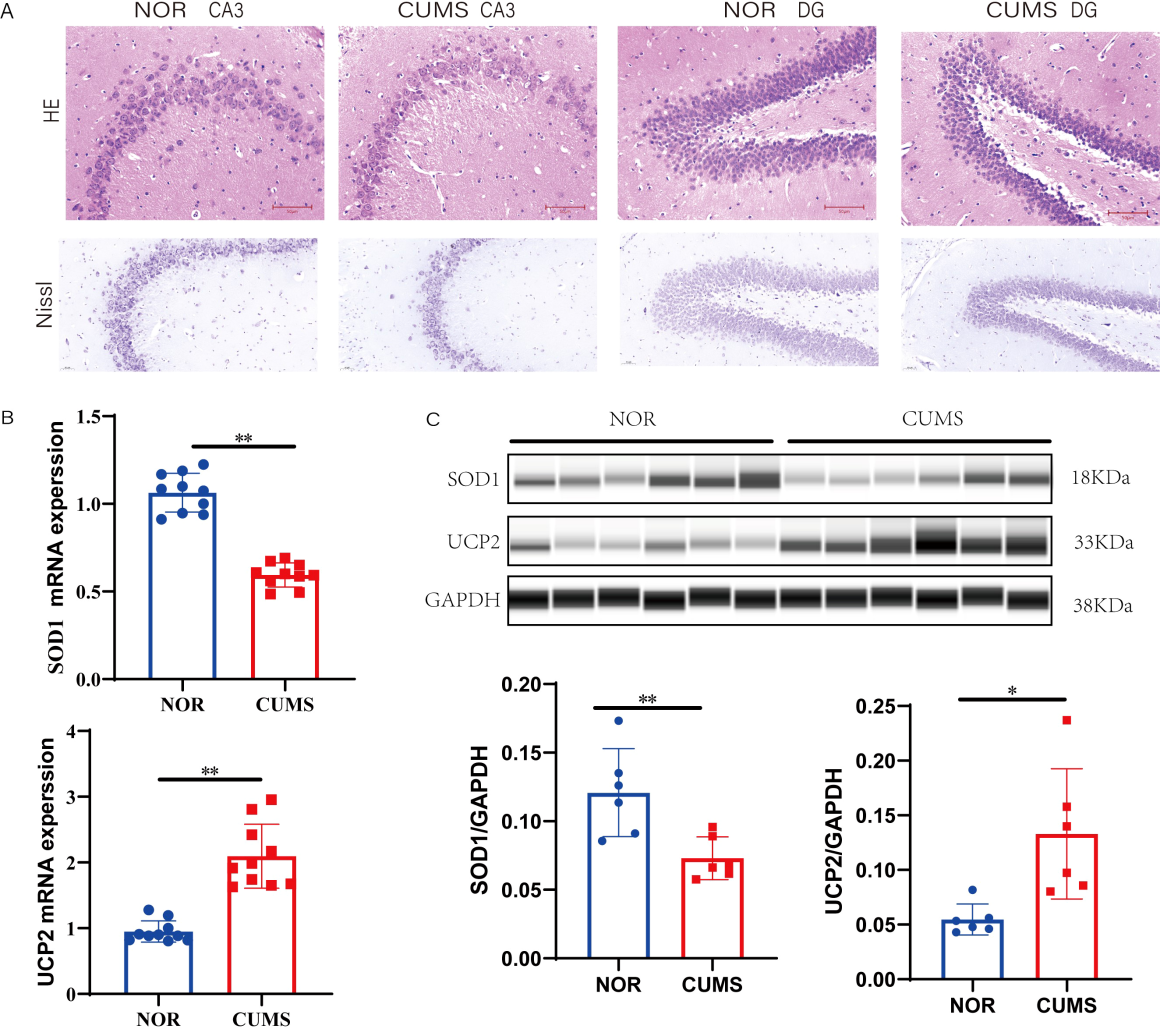
Validation of histological injury and risk gene expression in the hippocampus. **(A)** Representative images of HE (top) and Nissl (bottom) staining in the CA3 and DG subregions. The CUMS group exhibited neuronal loss and nuclear pyknosis compared to controls. Scale bar = 50 μm. **(B)** Relative mRNA levels of SOD1 and UCP2 analyzed by RT-qPCR. **(C)** Western blot analysis and densitometric quantification of SOD1 and UCP2 protein expression normalized to GAPDH. Data are presented as mean ± SD. Unpaired Student’s t-test. *P < 0.05, **P < 0.01 vs. NOR group.

Next, we assessed neuropathological changes in the hippocampus. Histopathological examination using H&E staining revealed significant neuronal injury in the CUMS group. In contrast to the neatly arranged and densely packed neurons in the CA3 and dentate gyrus (DG) regions of control animals, the CUMS group exhibited cellular disarray, reduced neuronal density, and enlarged intercellular spaces (**Figure 10A**). At higher magnification, neurons from CUMS rats displayed hallmarks of cellular stress, including chromatolysis (loss of Nissl bodies) and pyknosis (nuclear condensation and shrinkage), whereas control neurons appeared healthy with abundant Nissl substance and intact nuclei.

Finally, we sought to determine whether the expression of key genes identified by our MR analysis was altered in the hippocampus of the CUMS model. We first examined *UCP2*, which the MR analysis implicated as a risk factor for MDD. Consistent with the human genetic data, both *UCP2* mRNA and protein levels were significantly upregulated in the hippocampus of CUMS rats relative to controls (*P* < 0.05, **Figures 10B, C**). Next, we assessed the expression of *SOD1*. Intriguingly, in contrast to the MR finding where higher *SOD1* expression in mononuclear cells was associated with increased MDD risk, we observed a significant downregulation of both *SOD1* mRNA and protein levels in the hippocampus of the CUMS model (*P* < 0.05, **Figures 10B, C**). Collectively, these *in vivo* findings validate the role of hippocampal *UCP2* upregulation in depressive-like pathology. However, the opposing expression pattern of *SOD1* between the CUMS model and human genetic data suggests a complex, potentially context-dependent role for this gene in MDD pathophysiology that warrants further investigation.

## 4 Discussion

In this study, we integrated multi-omics data with Mendelian randomization to dissect the causal role of mitochondrial immunometabolism in MDD. The primary contributions of our work are fourfold. First, we developed a novel integrative framework that uses machine learning and single-cell genomics to prioritize causally-relevant genes, moving beyond simple correlation. Second, we provide the first genetic evidence for a causal link between the expression of five specific genes (*HK2*, *NDUFS4*, *NEU1*, *SOD1*, and *UCP2*) in distinct immune cell types and the risk of MDD, identifying both risk and protective profiles. Third, we established the clinical potential of this five-gene signature as a diagnostic biomarker panel, demonstrating good performance in an independent cohort. Finally, we experimentally corroborated our genetic findings by observing the upregulation of the principal risk gene, *UCP2*, in the hippocampus of a stress-induced rat model. Collectively, these findings illuminate a novel immune-metabolic axis in depression and highlight genetically prioritized targets for future therapeutic development.

Our single-cell eQTL-based MR analysis provided granular insights into the cell-type-specific causal architecture of these genes. The protective gene *HK2*, whose higher expression in monocytes was associated with reduced MDD risk, demonstrated fair diagnostic utility (AUC = 0.684). Specifically, our MR results confirmed that genetically predicted upregulation of *HK2* in monocytes exerts a robust protective effect against MDD. This finding presents an intriguing paradox. While literature suggests its upregulation often drives pro-inflammatory aerobic glycolysis (the Warburg effect) (35, 36), our data suggest its role in monocytes is protective. This could imply that a baseline level of glycolytic readiness is essential for proper monocyte function, and that genetic variants promoting this optimal state are protective, whereas dysregulated glycolysis contributes to pathology. Similarly, we MR results identified protective roles for *NDUFS4* (AUC = 0.647) in CD4+ naive T cells and natural killer cells, and *NEU1* (AUC = 0.602) in CD4+ naive T cells were all causally associated with a reduced risk of MDD. *NDUFS4* is vital for mitochondrial Complex I efficiency (37) and *NEU1* is a key lysosomal enzyme (38), underscoring that preserving fundamental energy and cellular maintenance processes within the immune system is a key factor in mitigating MDD risk. We hypothesize that *NEU1* may confer protection by regulating the sialylation of key surface receptors on T cells, thereby modulating activation, differentiation, and migration processes (39). Dysregulation of these processes has been implicated in neuroinflammation, suggesting that *NEU1* may help maintain immune homeostasis and prevent T cell-mediated pathology relevant to MDD (40, 41).

In stark contrast, our findings nominate *UCP2* as a central, multi-lineage driver of MDD pathology. Increased *UCP2* expression emerged as a consistent causal risk factor across a broad spectrum of immune cells and showed fair diagnostic potential (AUC = 0.635). This aligns perfectly with the concept of maladaptive immunometabolic reprogramming in chronic inflammatory states linked to MDD (9, 42). As a key modulator of mitochondrial function and ROS production (43, 44), *UCP2* upregulation in immune cells likely signifies a state of chronic metabolic stress that fuels the low-grade neuroinflammation characteristic of depression (45). Our *in vivo* results provide experimental support for this hypothesis: we observed significantly elevated mRNA and protein levels of *UCP2* in the hippocampus of CUMS rats. This finding is consistent with previous studies linking *UCP2* to neurological disorders and depressive-like phenotypes (46). The convergence of human genetic evidence and *in vivo* data reinforces *UCP2* as a promising therapeutic target for MDD.

Our findings on the context-dependent role of *SOD1* provide compelling evidence to reconcile conflicting reports in the literature regarding its involvement in MDD. It is well-established that *SOD1* exerts a neuroprotective role in the central nervous system (CNS) (47). Previous studies have reported lower *SOD1* levels in MDD patients and have shown that *SOD1* overexpression can protect rodents from stress-induced depressive-like behaviors, likely by mitigating oxidative stress (48, 49). Consistent with this neuroprotective paradigm, our CUMS model revealed a significant downregulation of *SOD1* in the hippocampus, suggesting an exhaustion of the brain’s antioxidant defenses under chronic stress. However, our MR analysis unveils a potentially paradoxical role for *SOD1* in the peripheral immune system. We found that a genetic predisposition for higher *SOD1* expression in immune cells may confer a risk factor for MDD. This seemingly paradoxical finding may reflect distinct context-dependent roles of *SOD1*. This leads us to hypothesize a dual, tissue-specific role for *SOD1*. In the CNS, its primary function is antioxidant defense, where higher levels are protective. In contrast, within immune cells, *SOD1* is a critical modulator of redox signaling, which governs inflammatory responses. Collectively, these observations highlight the complexity of targeting *SOD1* and underscore the imperative for tissue-specific therapeutic strategies in depression management.

Crucially, while our MR analysis primarily focused on peripheral immune cells, it is important to acknowledge that mitochondrial dysfunction is not confined to the periphery but may also manifest in CNS glial cells, particularly microglia and astrocytes (50, 51). These glial cells are pivotal for maintaining neuronal homeostasis and regulating neuroinflammation, processes that are fundamentally dependent on mitochondrial integrity. We propose that aberrant expression of mitochondrial metabolic genes in peripheral immune cells could propagate signals (via cytokines or extracellular vesicles) to the CNS, thereby influencing glial metabolism and activity. This communication suggests a potential “peripheral-central immune-glial mitochondrial axis” contributing to the immunometabolic imbalance in MDD. In the current study, we primarily validated the pathological alterations of *UCP2* and *SOD1* in the hippocampal tissue of CUMS rats. Future investigations utilizing single-cell transcriptomics are warranted to specifically delineate the cell-type-specific roles of these genes within CNS glial populations, further refining the immunometabolic model of MDD.

The six genes that showed initial correlation but lacked causal evidence in our MR analysis (*ATP7B*, *CRAT, GRIA1, LIPT1, MAP2K6, TNNT1*) are also informative. Their associations may be secondary to the disease process, influenced by confounding variables, or represent pathways that are not central to disease etiology. For instance, while altered copper metabolism (*ATP7B*) has been linked to depression (52, 53), our results suggest it may not be a primary causal driver. This distinction powerfully illustrates the discriminatory power of MR in prioritizing targets with genuine causal impact from a pool of observational associations, saving resources for downstream functional studies (54). These results align with broader MDD research, including Psychiatric Genomics Consortium GWAS implicating immune and neuronal genes (55). Our study adds a layer of causal evidence, suggesting that genetic variants affecting the expression levels of specific genes in immune cells are key drivers of this risk. This high-precision causal mapping offers a new level of clarity for prioritizing genetically-validated targets for future mechanism-based immunomodulatory therapies.

Despite the robustness of our multi-pronged approach, several limitations should be acknowledged. First, although sensitivity analyses suggest robust causal inference, the inherent possibility of horizontal pleiotropy in MR cannot be definitively excluded. Second, the predominance of European ancestry in the GWAS datasets may limit generalizability, necessitating verification in diverse populations. Finally, our current *in vivo* validation relies on bulk tissue analysis, which confirms overall dysregulation but lacks the resolution to distinguish specific immune or glial sources. Future work utilizing spatial transcriptomics or multiplex imaging is warranted to spatially map these cell-type-specific effects and elucidate their downstream mechanisms.

## 5 Conclusions

In conclusion, this study provides compelling, multi-faceted evidence that the expression of five key mitochondria-related genes in specific immune cells exerts a causal effect on MDD risk. We established their potential as clinically relevant biomarkers, verified the upregulation of the risk gene *UCP2* in a stress-induced animal model, and uncovered a complex, context-dependent role for *SOD1*. These findings move beyond correlation to illuminate a distinct immune-metabolic axis in depression. We propose the identified pathways as genetically supported candidates for the development of novel mechanism-based diagnostics and immunomodulatory therapies for MDD.

## Data Availability

The datasets presented in this study can be found in online repositories. The names of the repository/repositories and accession number(s) can be found in the article/**Supplementary Material**.
