# Supplementary material for: Single-cell transcriptome-wide Mendelian randomization identifies mitochondrial targets in immune cells for Major Depressive Disorder
Source: Front Immunol. 2026 Jan 12;16:1700604. doi: 10.3389/fimmu.2025.1700604 (PMC12832325; doi:10.3389/fimmu.2025.1700604)
Supplement: Supplementary file 1 [file DataSheet1.docx]

**Supplementary Methods & Figures Overview**

This Supplementary Information provides detailed descriptions of additional methods and sensitivity analyses performed to ensure the robustness of our Mendelian randomization (MR) findings on the causal effects of immune cell–specific gene expression on major depressive disorder (MDD) risk. In addition to the primary two-sample MR analyses reported in the main text, we conducted a comprehensive set of complementary tests—including MR-Egger regression, weighted median/mode estimators, Cochran’s Q test, MR-PRESSO global test, and leave-one-out analyses—to evaluate the validity of the instrumental variables and to assess potential heterogeneity and horizontal pleiotropy.

The supplementary figures (Figures S1–S16) provide graphical summaries of these sensitivity analyses. Forest plots and scatter plots illustrate causal estimates across different MR methods and SNP-specific effects, funnel plots evaluate the symmetry of effect distributions, and leave-one-out plots demonstrate the stability of causal estimates when excluding individual variants. Collectively, these analyses confirmed that the reported associations were consistent, free from substantial pleiotropy, and not driven by single outlier SNPs.

By integrating these supplementary methods and figures with the main results, the present work provides a rigorous and transparent assessment of the causal contributions of gene expression in specific immune cell types to MDD pathogenesis.


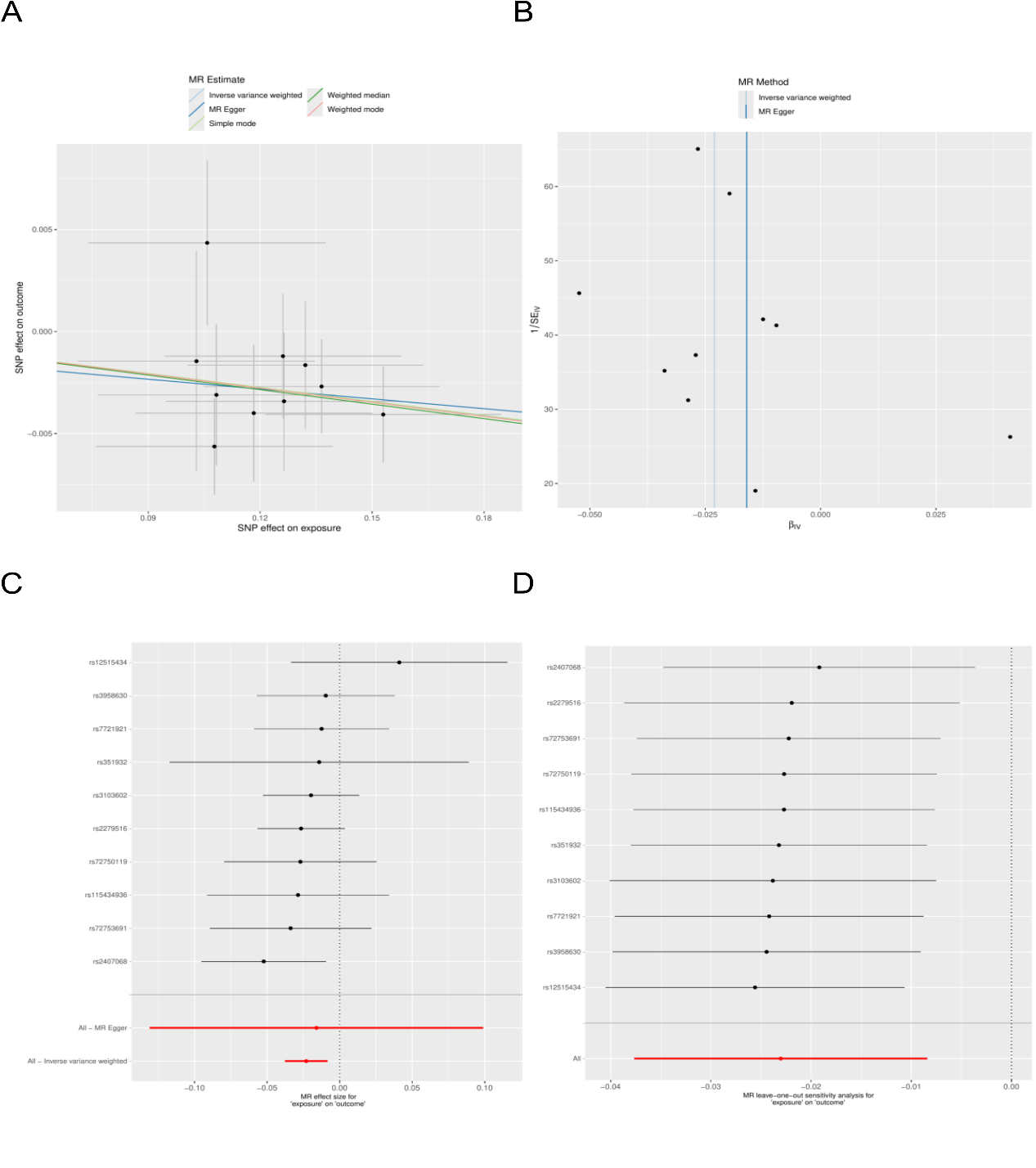


**Supplementary Figure S1. Sensitivity analyses for the causal association between *NDUFS4* expression in cd4nc and MDD risk.**

(A) Scatter plot illustrating the causal effect of *NDUFS4* expression on MDD. Each point represents a single instrumental SNP. The slope of the regression line for each MR method corresponds to the estimated causal effect.

(B) Funnel plot visualizing the distribution of single-SNP effects against their precision. The symmetrical distribution of SNPs around the summary estimate suggests an absence of directional pleiotropy.

(C) Forest plot showing the causal effect estimated by each individual SNP (Single Nucleotide Polymorphism) alongside the combined estimates from the IVW and MR-Egger methods.

(D) Leave-one-out sensitivity analysis. Each point represents the overall MR estimate after removing that particular SNP from the analysis. The results indicate that no single SNP was overly influential on the final causal estimate.


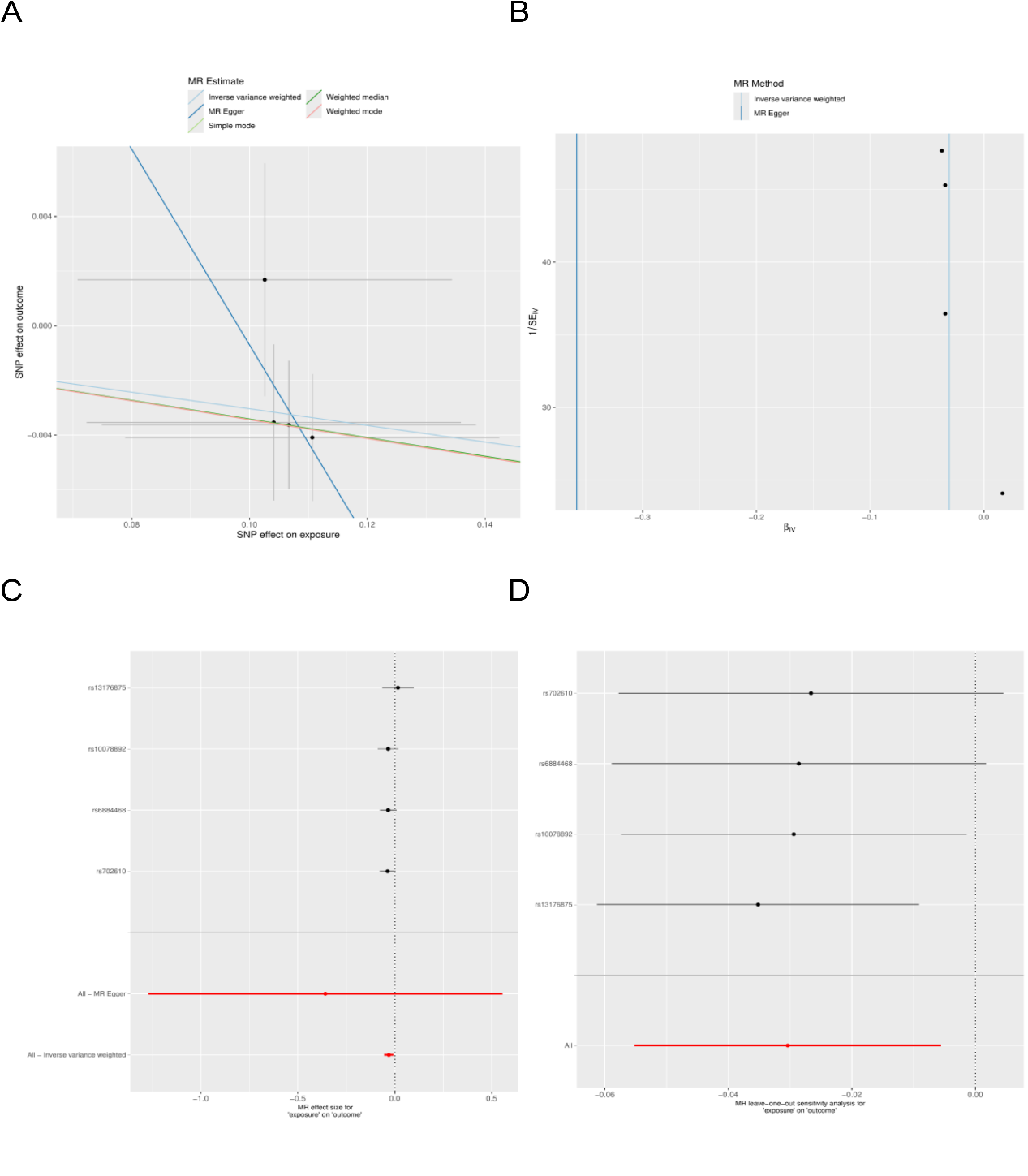


**Supplementary Figure S2. Sensitivity analyses for the causal association between *NDUFS4* expression in nk and MDD risk.**

(A) Scatter plot illustrating the causal effect of *NDUFS4* expression on MDD. Each point represents a single instrumental SNP. The slope of the regression line for each MR method corresponds to the estimated causal effect.

(B) Funnel plot visualizing the distribution of single-SNP effects against their precision. The symmetrical distribution of SNPs around the summary estimate suggests an absence of directional pleiotropy.

(C) Forest plot showing the causal effect estimated by each individual SNP (Single Nucleotide Polymorphism) alongside the combined estimates from the IVW and MR-Egger methods.

(D) Leave-one-out sensitivity analysis. Each point represents the overall MR estimate after removing that particular SNP from the analysis. The results indicate that no single SNP was overly influential on the final causal estimate.


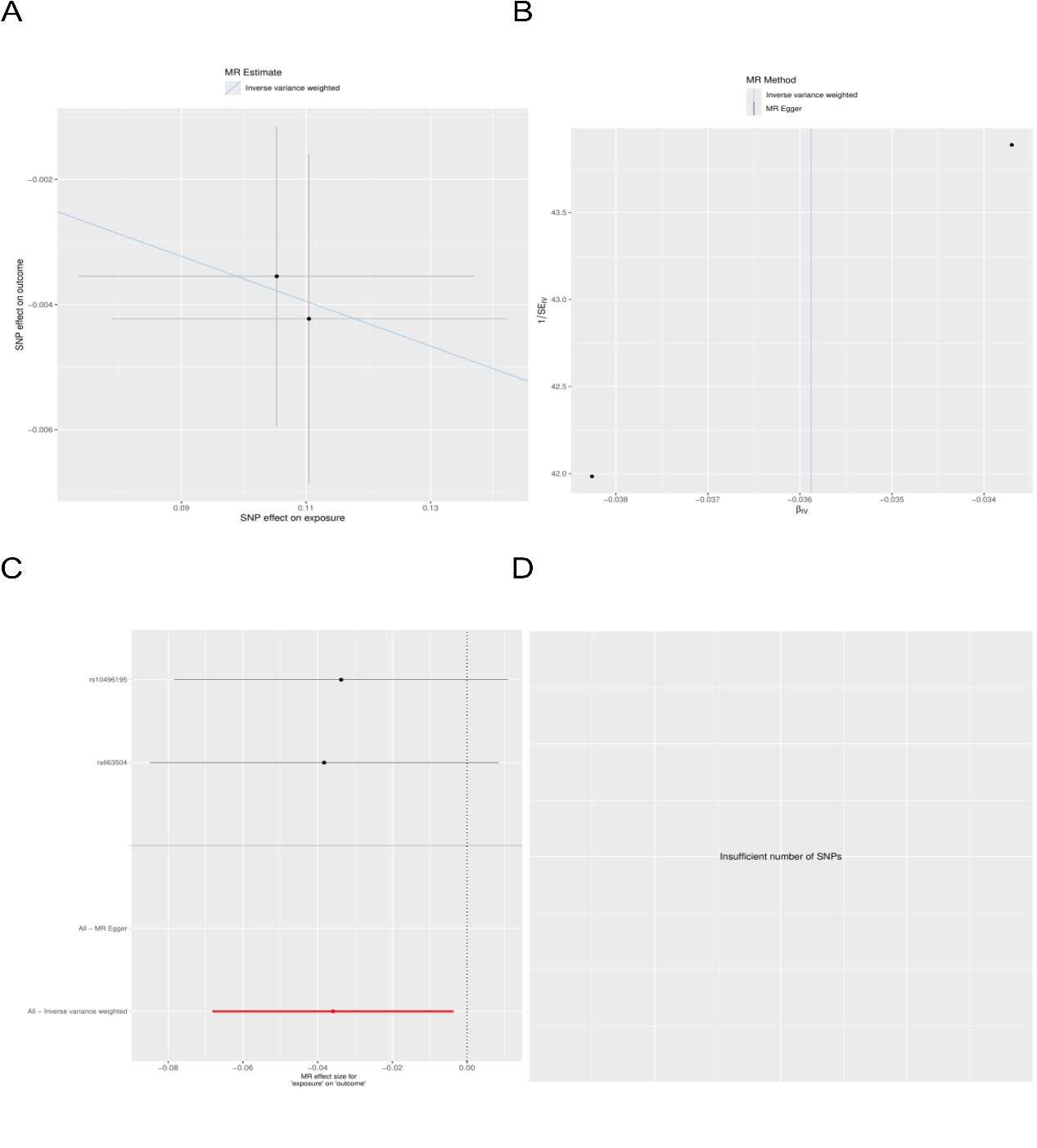


**Supplementary Figure S3. Sensitivity analyses for the causal association between *HK2* expression in monoc and MDD risk.**

(A) Scatter plot illustrating the causal effect of *HK2* expression on MDD. Each point represents a single instrumental SNP. The slope of the regression line for each MR method corresponds to the estimated causal effect.

(B) Funnel plot visualizing the distribution of single-SNP effects against their precision. The symmetrical distribution of SNPs around the summary estimate suggests an absence of directional pleiotropy.

(C) Forest plot showing the causal effect estimated by each individual SNP (Single Nucleotide Polymorphism) alongside the combined estimates from the IVW and MR-Egger methods.

(D) Leave-one-out sensitivity analysis. Each point represents the overall MR estimate after removing that particular SNP from the analysis. The results indicate that no single SNP was overly influential on the final causal estimate.

**
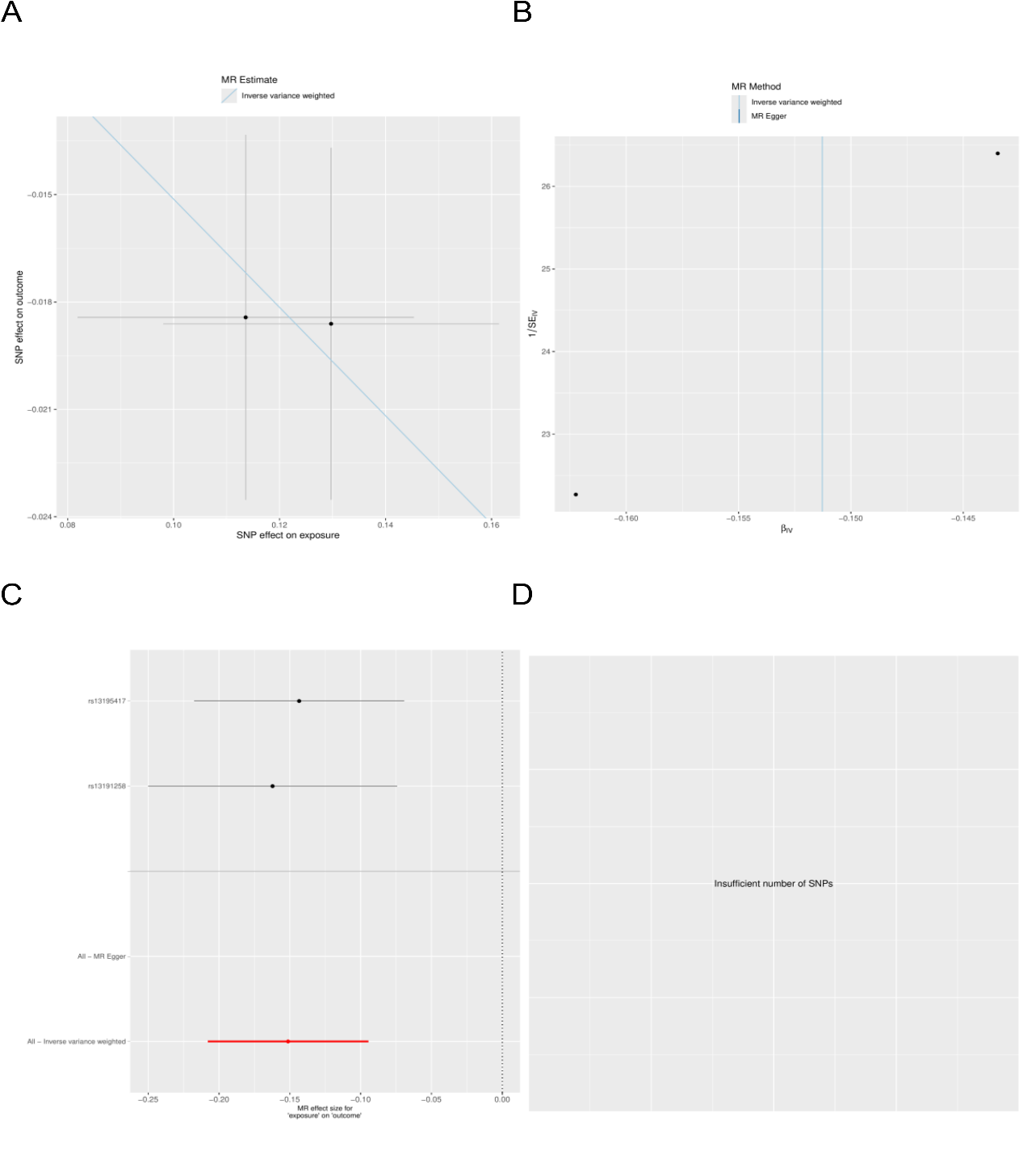
**

**Supplementary Figure S4. Sensitivity analyses for the causal association between *NEU1* expression in** **cd4nc and MDD risk.**

(A) Scatter plot illustrating the causal effect of *NEU1* expression on MDD. Each point represents a single instrumental SNP. The slope of the regression line for each MR method corresponds to the estimated causal effect.

(B) Funnel plot visualizing the distribution of single-SNP effects against their precision. The symmetrical distribution of SNPs around the summary estimate suggests an absence of directional pleiotropy.

(C) Forest plot showing the causal effect estimated by each individual SNP (Single Nucleotide Polymorphism) alongside the combined estimates from the IVW and MR-Egger methods.

(D) Leave-one-out sensitivity analysis. Each point represents the overall MR estimate after removing that particular SNP from the analysis. The results indicate that no single SNP was overly influential on the final causal estimate.

**
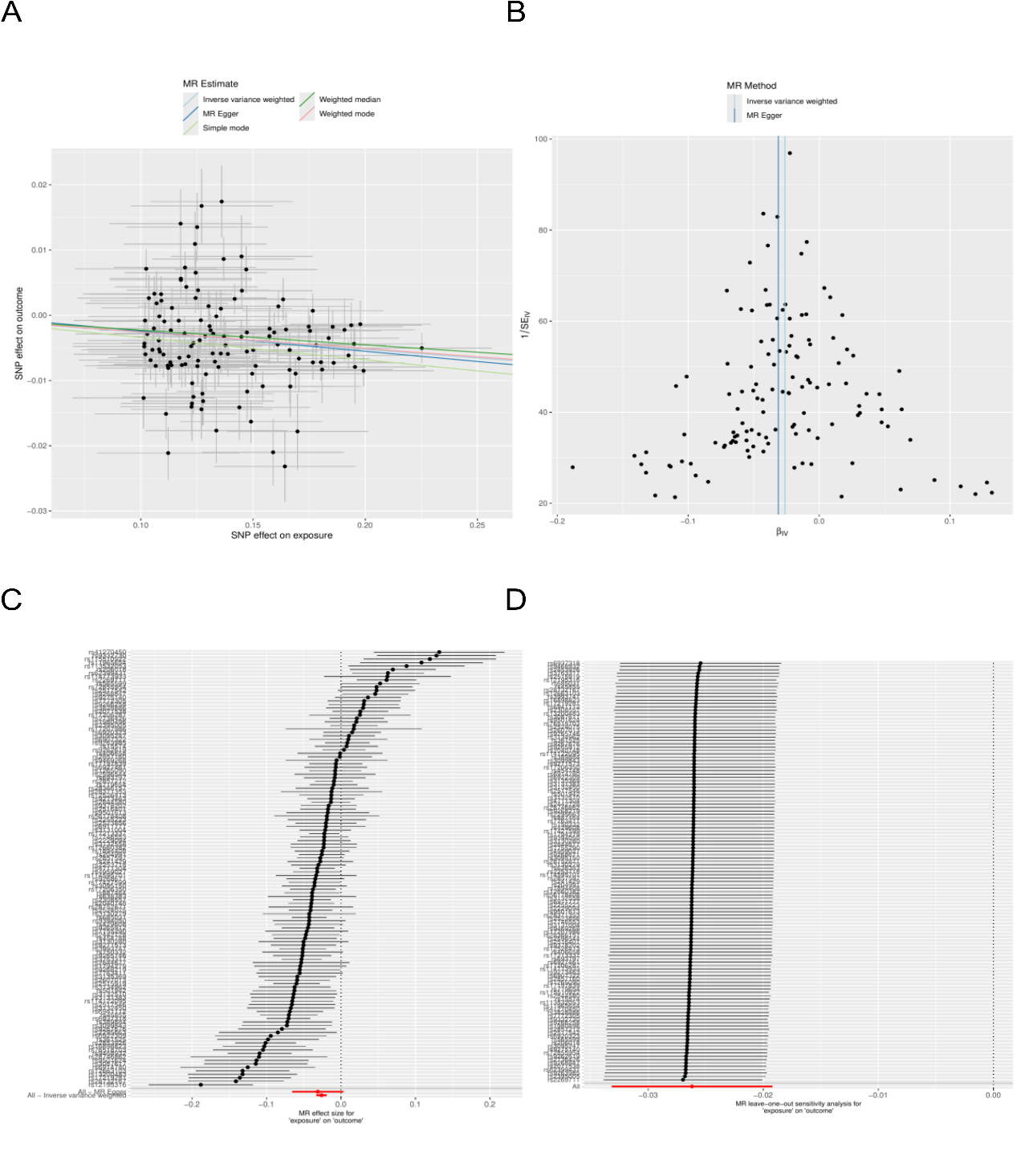
**

**Supplementary Figure S5. Sensitivity analyses for the causal association between *NEU1* expression in** **cd4sox4 and MDD risk.**

(A) Scatter plot illustrating the causal effect of *NEU1* expression on MDD. Each point represents a single instrumental SNP. The slope of the regression line for each MR method corresponds to the estimated causal effect.

(B) Funnel plot visualizing the distribution of single-SNP effects against their precision. The symmetrical distribution of SNPs around the summary estimate suggests an absence of directional pleiotropy.

(C) Forest plot showing the causal effect estimated by each individual SNP (Single Nucleotide Polymorphism) alongside the combined estimates from the IVW and MR-Egger methods.

(D) Leave-one-out sensitivity analysis. Each point represents the overall MR estimate after removing that particular SNP from the analysis. The results indicate that no single SNP was overly influential on the final causal estimate.

**
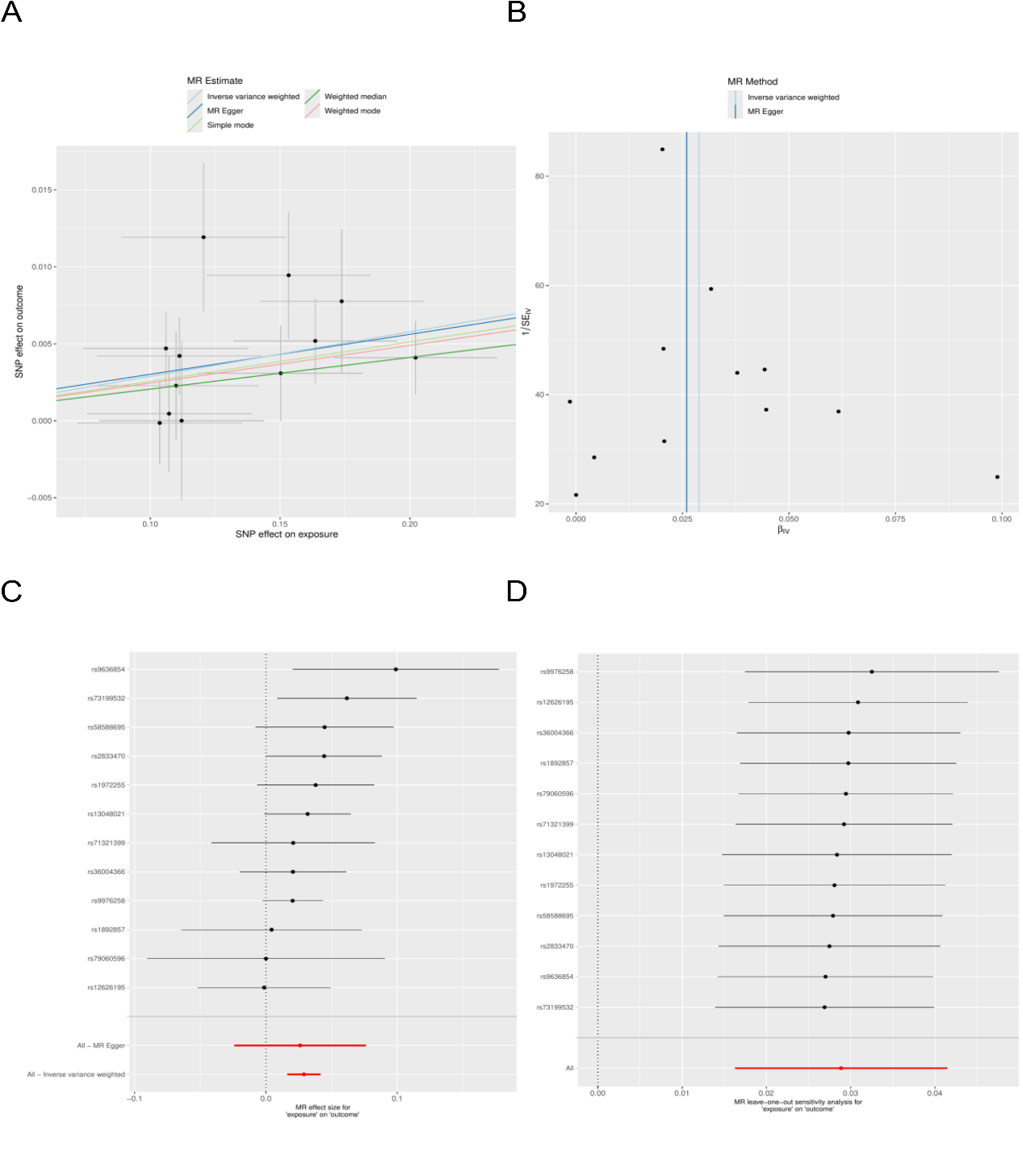
**

**Supplementary Figure S6. Sensitivity analyses for the causal association between *SOD1* expression in** **mononc and MDD risk.**

(A) Scatter plot illustrating the causal effect of *SOD1* expression on MDD. Each point represents a single instrumental SNP. The slope of the regression line for each MR method corresponds to the estimated causal effect.

(B) Funnel plot visualizing the distribution of single-SNP effects against their precision. The symmetrical distribution of SNPs around the summary estimate suggests an absence of directional pleiotropy.

(C) Forest plot showing the causal effect estimated by each individual SNP (Single Nucleotide Polymorphism) alongside the combined estimates from the IVW and MR-Egger methods.

(D) Leave-one-out sensitivity analysis. Each point represents the overall MR estimate after removing that particular SNP from the analysis. The results indicate that no single SNP was overly influential on the final causal estimate.

**
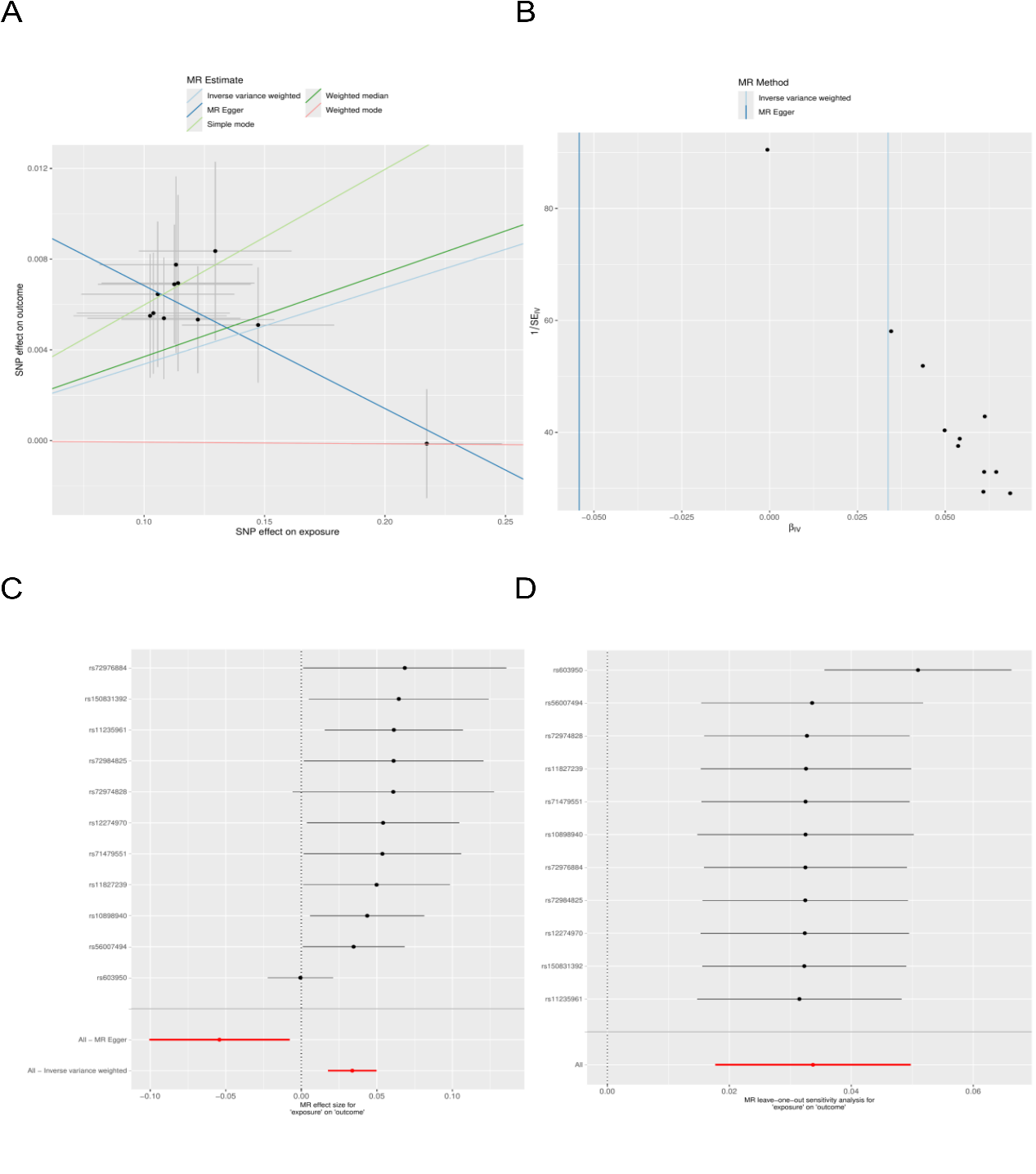
**

**Supplementary Figure S7. Sensitivity analyses for the causal association between *UCP2* expression in** **bin and MDD risk.**

(A) Scatter plot illustrating the causal effect of *UCP2* expression on MDD. Each point represents a single instrumental SNP. The slope of the regression line for each MR method corresponds to the estimated causal effect.

(B) Funnel plot visualizing the distribution of single-SNP effects against their precision. The symmetrical distribution of SNPs around the summary estimate suggests an absence of directional pleiotropy.

(C) Forest plot showing the causal effect estimated by each individual SNP (Single Nucleotide Polymorphism) alongside the combined estimates from the IVW and MR-Egger methods.

(D) Leave-one-out sensitivity analysis. Each point represents the overall MR estimate after removing that particular SNP from the analysis. The results indicate that no single SNP was overly influential on the final causal estimate.

**
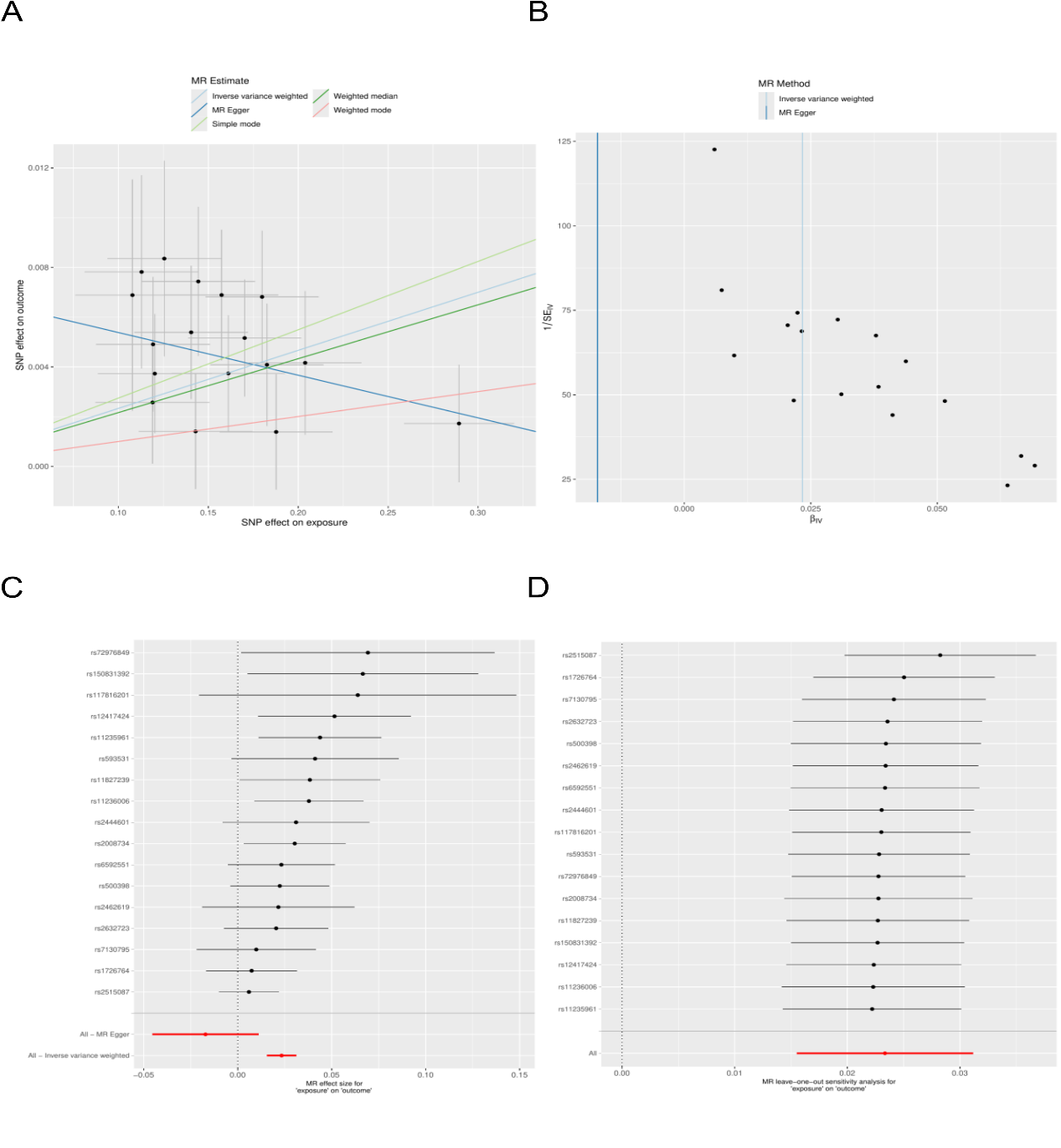
**

**Supplementary Figure S8. Sensitivity analyses for the causal association between *UCP2* expression in** **bmem and MDD risk.**

(A) Scatter plot illustrating the causal effect of *UCP2* expression on MDD. Each point represents a single instrumental SNP. The slope of the regression line for each MR method corresponds to the estimated causal effect.

(B) Funnel plot visualizing the distribution of single-SNP effects against their precision. The symmetrical distribution of SNPs around the summary estimate suggests an absence of directional pleiotropy.

(C) Forest plot showing the causal effect estimated by each individual SNP (Single Nucleotide Polymorphism) alongside the combined estimates from the IVW and MR-Egger methods.

(D) Leave-one-out sensitivity analysis. Each point represents the overall MR estimate after removing that particular SNP from the analysis. The results indicate that no single SNP was overly influential on the final causal estimate.

**
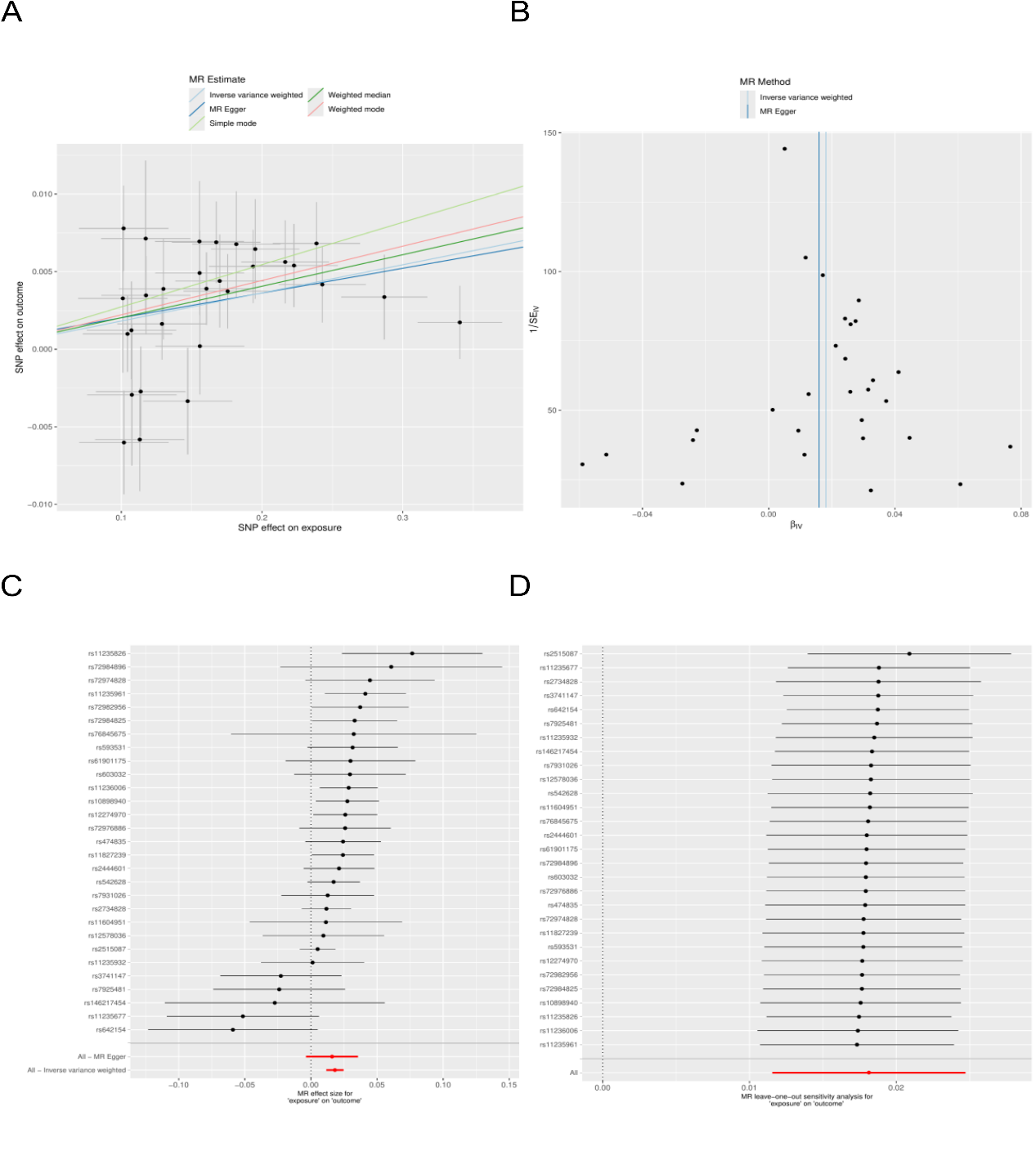
**

**Supplementary Figure S8. Sensitivity analyses for the causal association between *UCP2* expression in** **cd4et and MDD risk.**

(A) Scatter plot illustrating the causal effect of *UCP2* expression on MDD. Each point represents a single instrumental SNP. The slope of the regression line for each MR method corresponds to the estimated causal effect.

(B) Funnel plot visualizing the distribution of single-SNP effects against their precision. The symmetrical distribution of SNPs around the summary estimate suggests an absence of directional pleiotropy.

(C) Forest plot showing the causal effect estimated by each individual SNP (Single Nucleotide Polymorphism) alongside the combined estimates from the IVW and MR-Egger methods.

(D) Leave-one-out sensitivity analysis. Each point represents the overall MR estimate after removing that particular SNP from the analysis. The results indicate that no single SNP was overly influential on the final causal estimate.

**
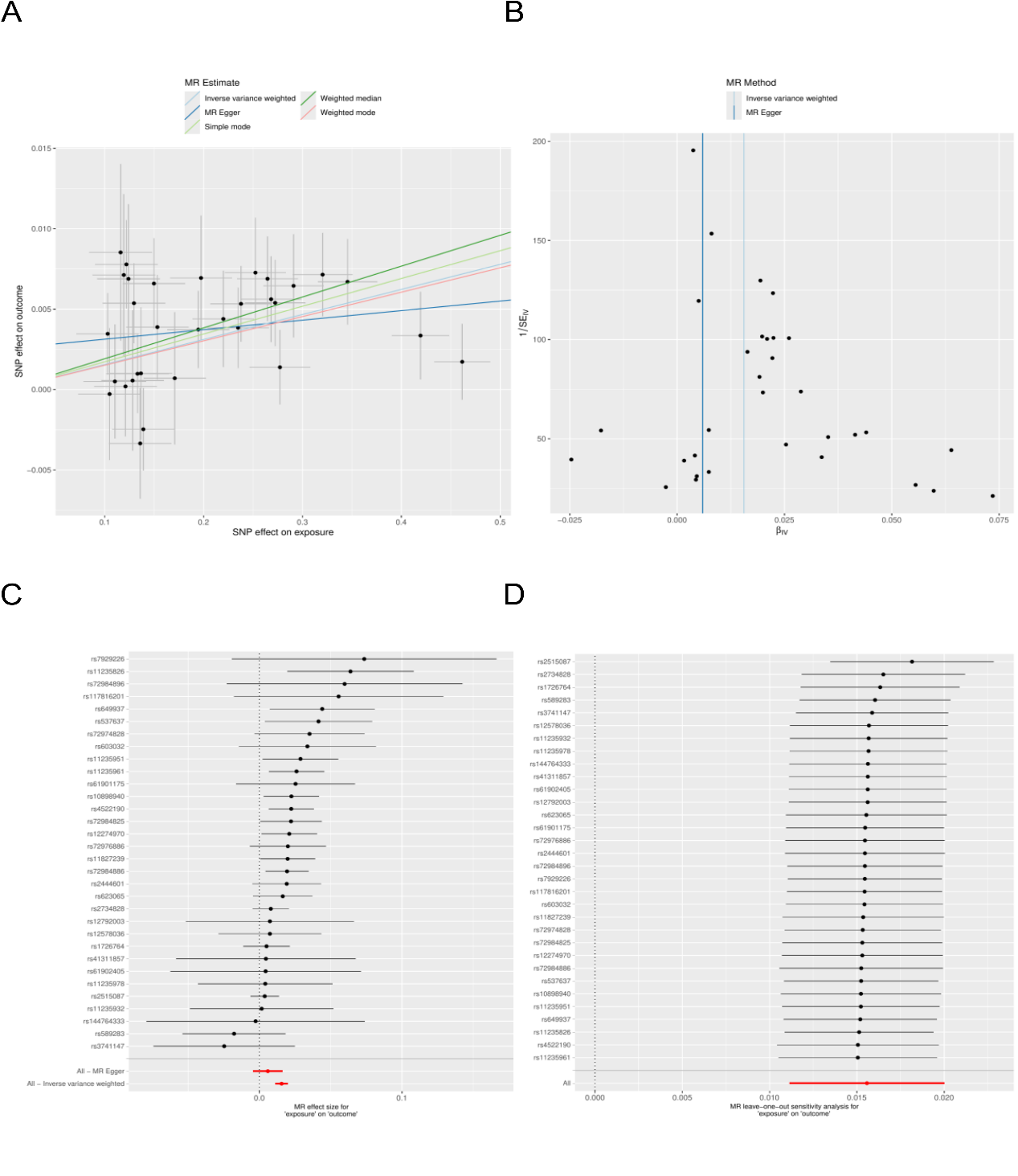
**

**Supplementary Figure S9. Sensitivity analyses for the causal association between *UCP2* expression in** **cd4nc and MDD risk.**

(A) Scatter plot illustrating the causal effect of *UCP2* expression on MDD. Each point represents a single instrumental SNP. The slope of the regression line for each MR method corresponds to the estimated causal effect.

(B) Funnel plot visualizing the distribution of single-SNP effects against their precision. The symmetrical distribution of SNPs around the summary estimate suggests an absence of directional pleiotropy.

(C) Forest plot showing the causal effect estimated by each individual SNP (Single Nucleotide Polymorphism) alongside the combined estimates from the IVW and MR-Egger methods.

(D) Leave-one-out sensitivity analysis. Each point represents the overall MR estimate after removing that particular SNP from the analysis. The results indicate that no single SNP was overly influential on the final causal estimate.

**
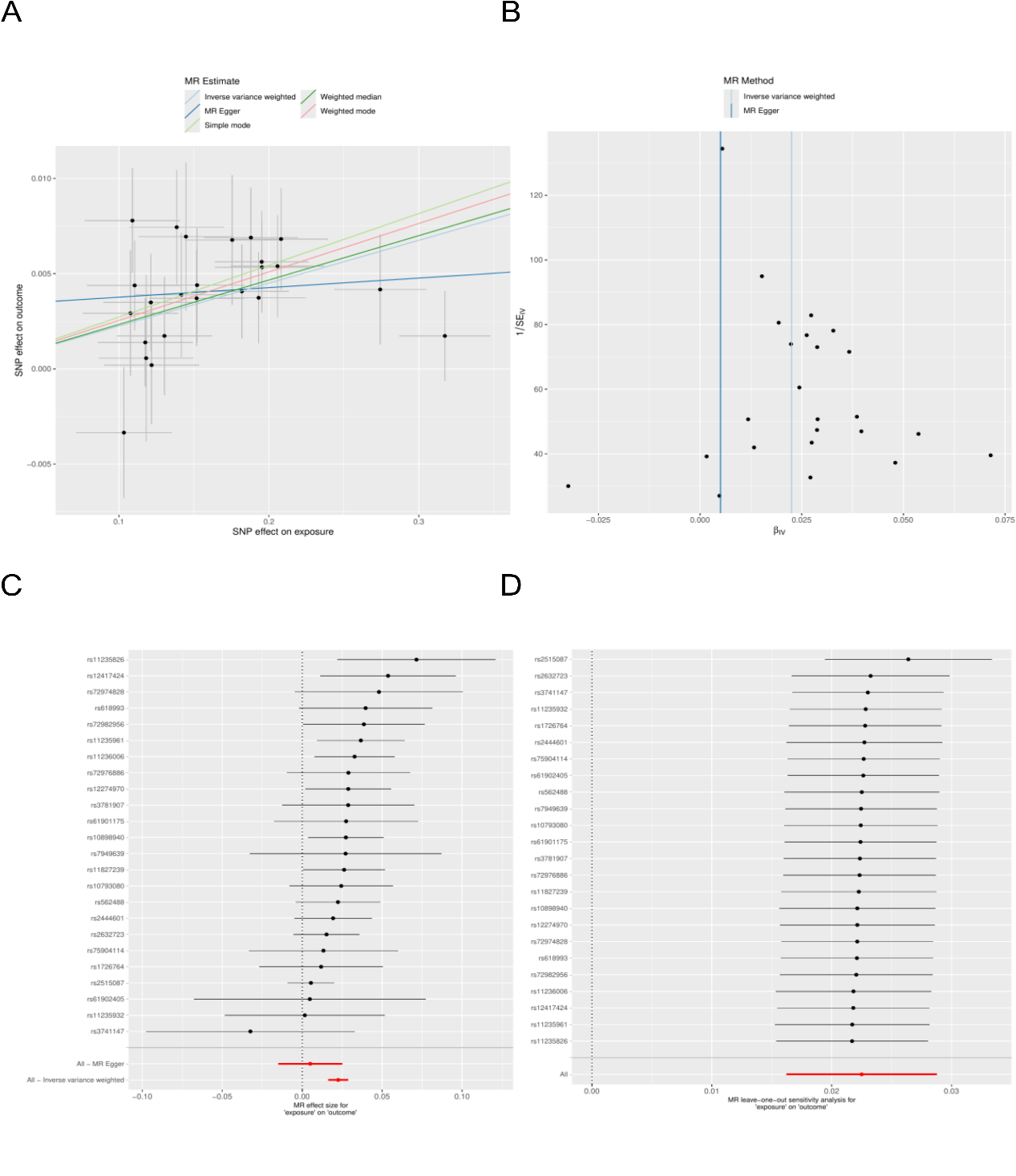
**

**Supplementary Figure S10. Sensitivity analyses for the causal association between *UCP2* expression in** **cd8et and MDD risk.**

(A) Scatter plot illustrating the causal effect of *UCP2* expression on MDD. Each point represents a single instrumental SNP. The slope of the regression line for each MR method corresponds to the estimated causal effect.

(B) Funnel plot visualizing the distribution of single-SNP effects against their precision. The symmetrical distribution of SNPs around the summary estimate suggests an absence of directional pleiotropy.

(C) Forest plot showing the causal effect estimated by each individual SNP (Single Nucleotide Polymorphism) alongside the combined estimates from the IVW and MR-Egger methods.

(D) Leave-one-out sensitivity analysis. Each point represents the overall MR estimate after removing that particular SNP from the analysis. The results indicate that no single SNP was overly influential on the final causal estimate.

**
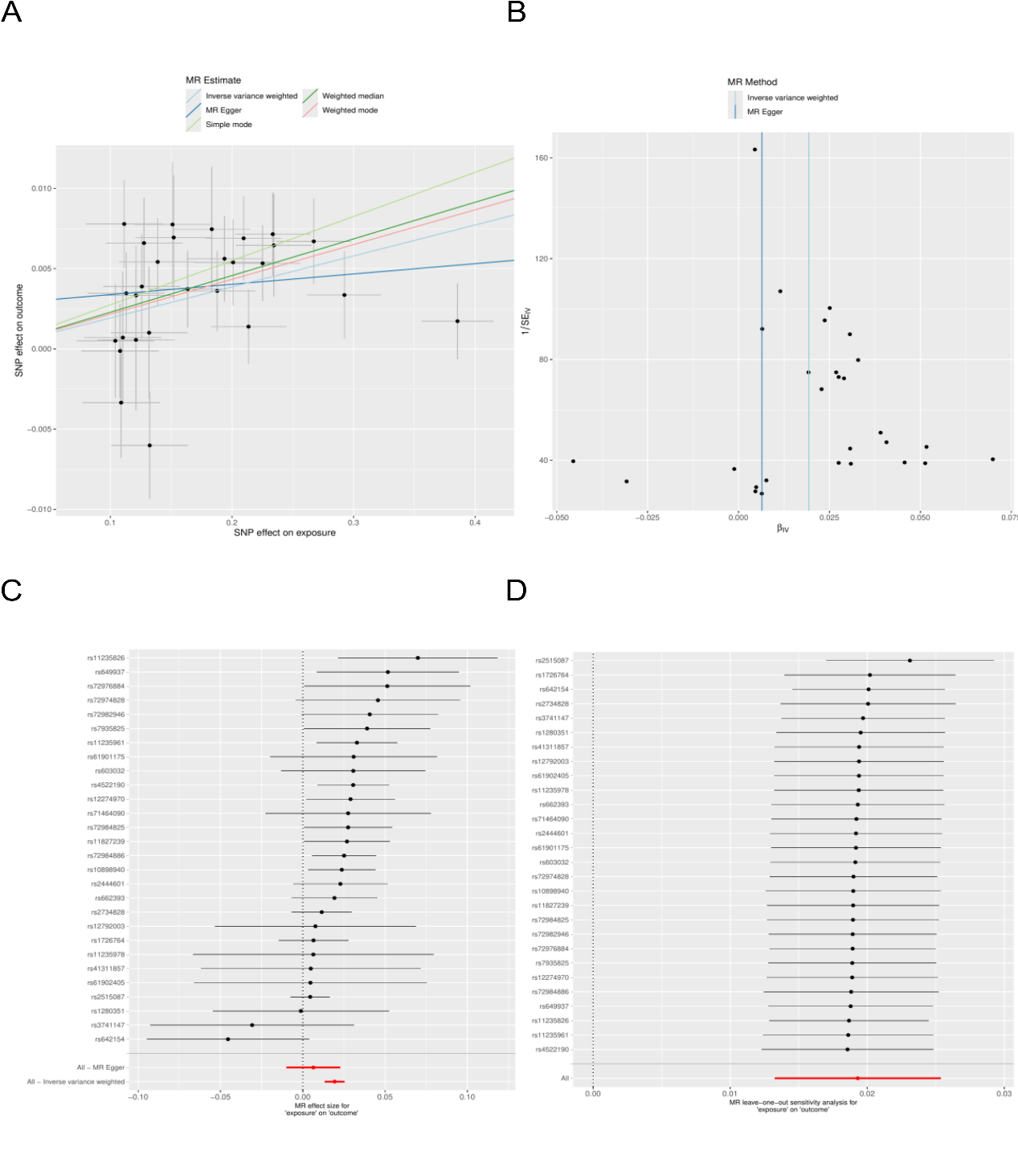
**

**Supplementary Figure S11. Sensitivity analyses for the causal association between *UCP2* expression in** **cd8nc and MDD risk.**

(A) Scatter plot illustrating the causal effect of *UCP2* expression on MDD. Each point represents a single instrumental SNP. The slope of the regression line for each MR method corresponds to the estimated causal effect.

(B) Funnel plot visualizing the distribution of single-SNP effects against their precision. The symmetrical distribution of SNPs around the summary estimate suggests an absence of directional pleiotropy.

(C) Forest plot showing the causal effect estimated by each individual SNP (Single Nucleotide Polymorphism) alongside the combined estimates from the IVW and MR-Egger methods.

(D) Leave-one-out sensitivity analysis. Each point represents the overall MR estimate after removing that particular SNP from the analysis. The results indicate that no single SNP was overly influential on the final causal estimate.

**
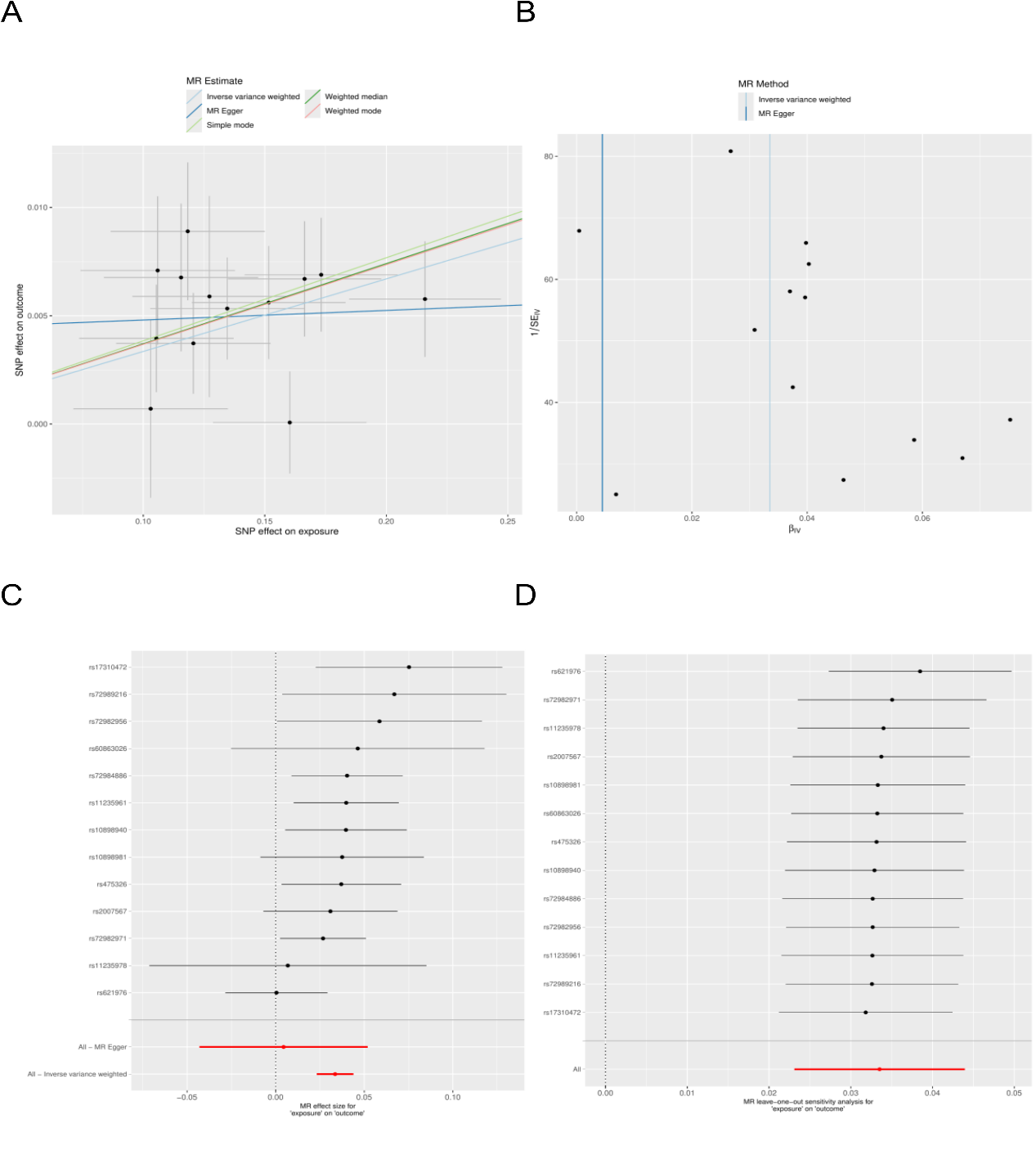
**

**Supplementary Figure S12. Sensitivity analyses for the causal association between *UCP2* expression in** **cd8s100b and MDD risk. dc**

(A) Scatter plot illustrating the causal effect of *UCP2* expression on MDD. Each point represents a single instrumental SNP. The slope of the regression line for each MR method corresponds to the estimated causal effect.

(B) Funnel plot visualizing the distribution of single-SNP effects against their precision. The symmetrical distribution of SNPs around the summary estimate suggests an absence of directional pleiotropy.

(C) Forest plot showing the causal effect estimated by each individual SNP (Single Nucleotide Polymorphism) alongside the combined estimates from the IVW and MR-Egger methods.

(D) Leave-one-out sensitivity analysis. Each point represents the overall MR estimate after removing that particular SNP from the analysis. The results indicate that no single SNP was overly influential on the final causal estimate.

**
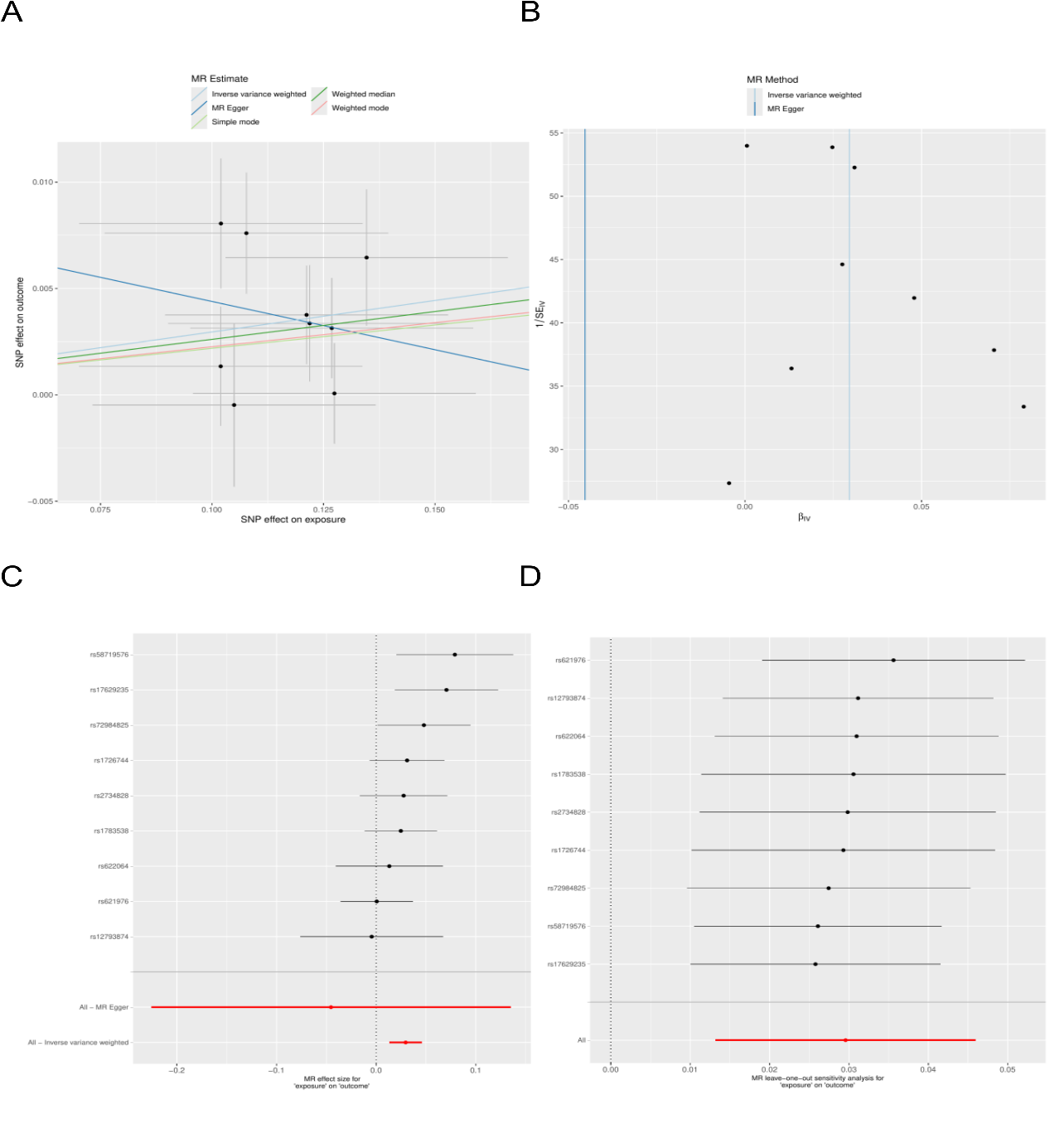
**

**Supplementary Figure S13. Sensitivity analyses for the causal association between *UCP2* expression in** **dc and MDD risk.**

(A) Scatter plot illustrating the causal effect of *UCP2* expression on MDD. Each point represents a single instrumental SNP. The slope of the regression line for each MR method corresponds to the estimated causal effect.

(B) Funnel plot visualizing the distribution of single-SNP effects against their precision. The symmetrical distribution of SNPs around the summary estimate suggests an absence of directional pleiotropy.

(C) Forest plot showing the causal effect estimated by each individual SNP (Single Nucleotide Polymorphism) alongside the combined estimates from the IVW and MR-Egger methods.

(D) Leave-one-out sensitivity analysis. Each point represents the overall MR estimate after removing that particular SNP from the analysis. The results indicate that no single SNP was overly influential on the final causal estimate.

**
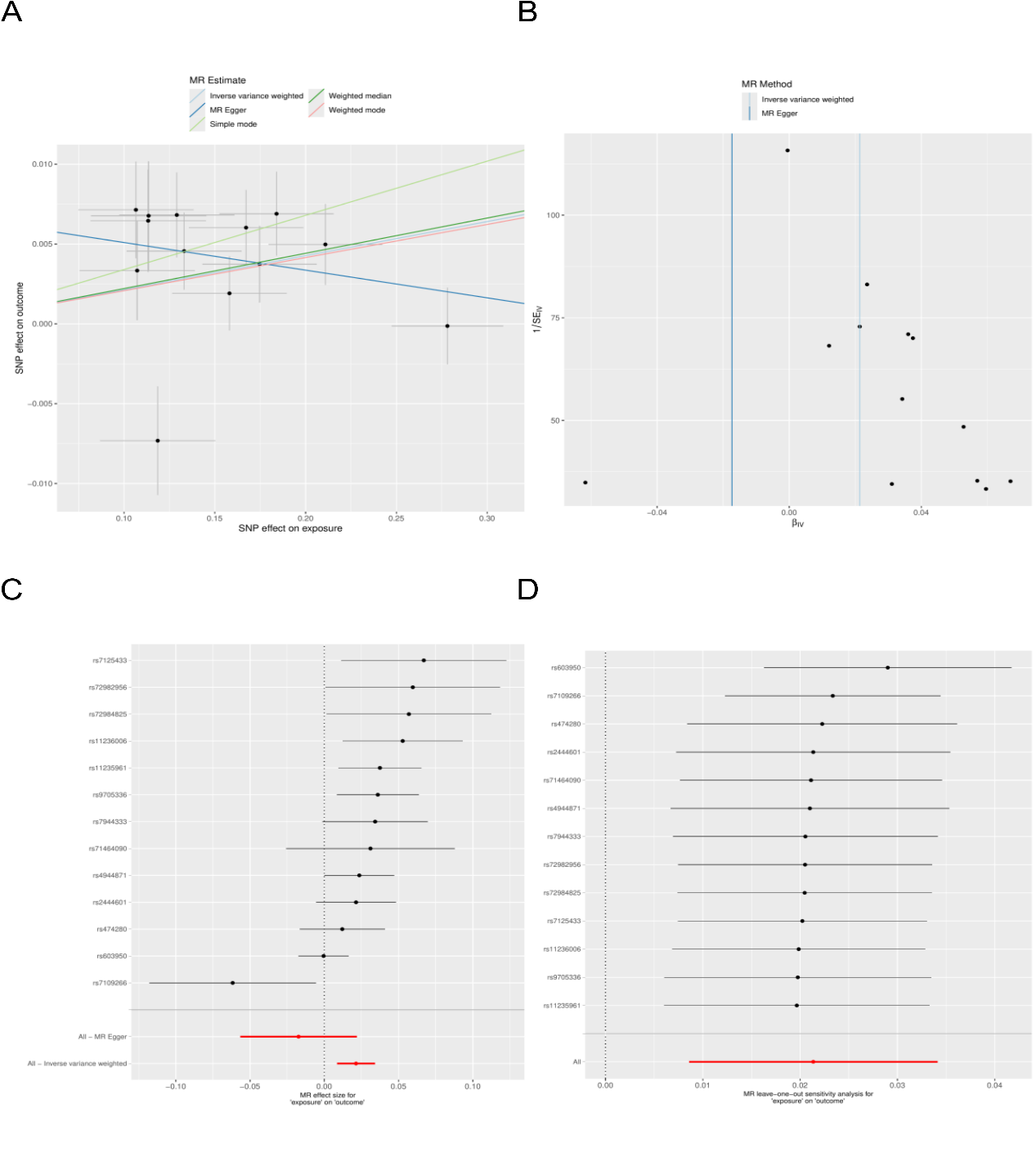
**

**Supplementary Figure S14. Sensitivity analyses for the causal association between *UCP2* expression in** **monoc and MDD risk.**

(A) Scatter plot illustrating the causal effect of *UCP2* expression on MDD. Each point represents a single instrumental SNP. The slope of the regression line for each MR method corresponds to the estimated causal effect.

(B) Funnel plot visualizing the distribution of single-SNP effects against their precision. The symmetrical distribution of SNPs around the summary estimate suggests an absence of directional pleiotropy.

(C) Forest plot showing the causal effect estimated by each individual SNP (Single Nucleotide Polymorphism) alongside the combined estimates from the IVW and MR-Egger methods.

(D) Leave-one-out sensitivity analysis. Each point represents the overall MR estimate after removing that particular SNP from the analysis. The results indicate that no single SNP was overly influential on the final causal estimate.

**
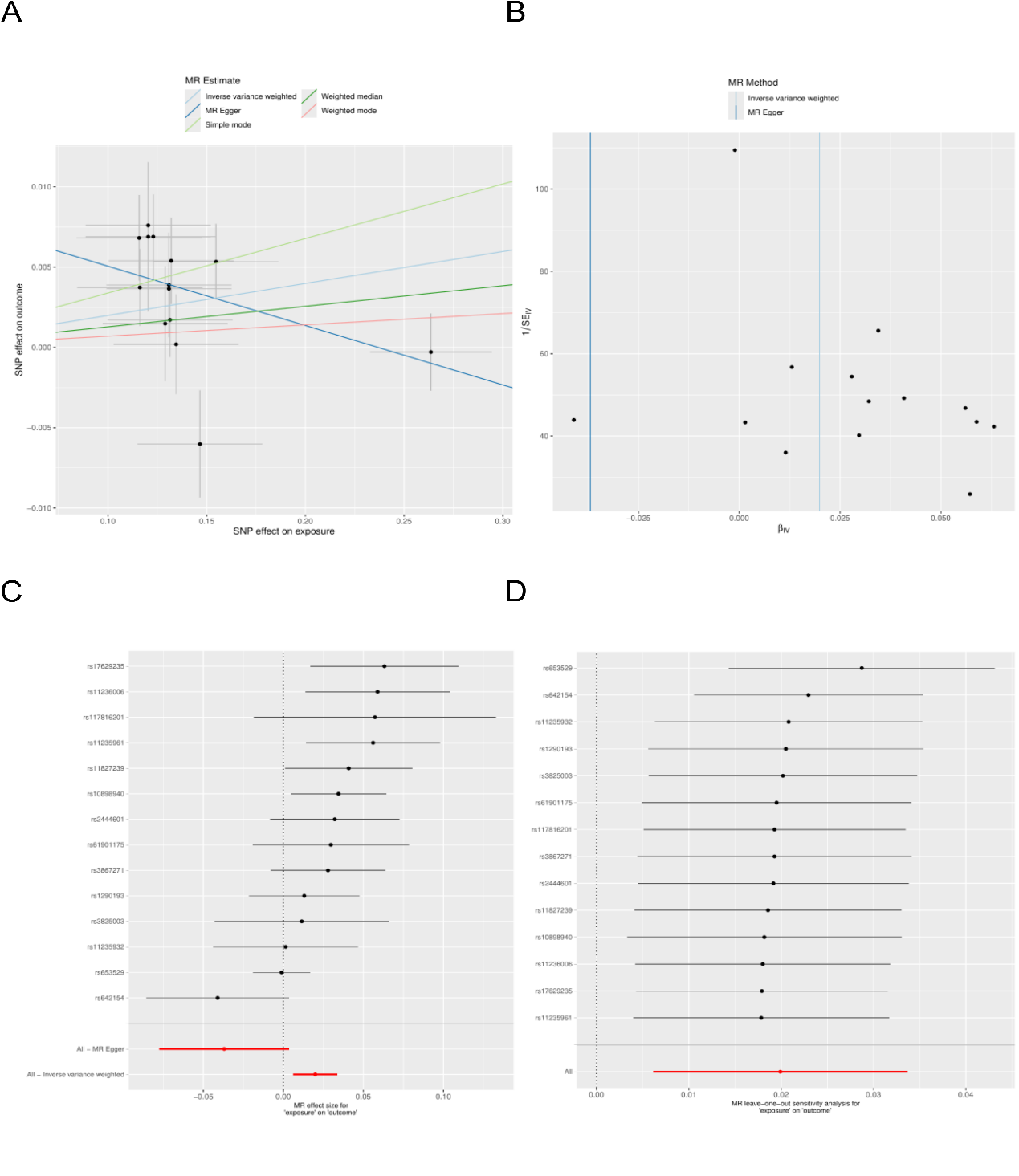
**

**Supplementary Figure S15. Sensitivity analyses for the causal association between *UCP2* expression in** **mononc and MDD risk.**

(A) Scatter plot illustrating the causal effect of *UCP2* expression on MDD. Each point represents a single instrumental SNP. The slope of the regression line for each MR method corresponds to the estimated causal effect.

(B) Funnel plot visualizing the distribution of single-SNP effects against their precision. The symmetrical distribution of SNPs around the summary estimate suggests an absence of directional pleiotropy.

(C) Forest plot showing the causal effect estimated by each individual SNP (Single Nucleotide Polymorphism) alongside the combined estimates from the IVW and MR-Egger methods.

(D) Leave-one-out sensitivity analysis. Each point represents the overall MR estimate after removing that particular SNP from the analysis. The results indicate that no single SNP was overly influential on the final causal estimate.

**
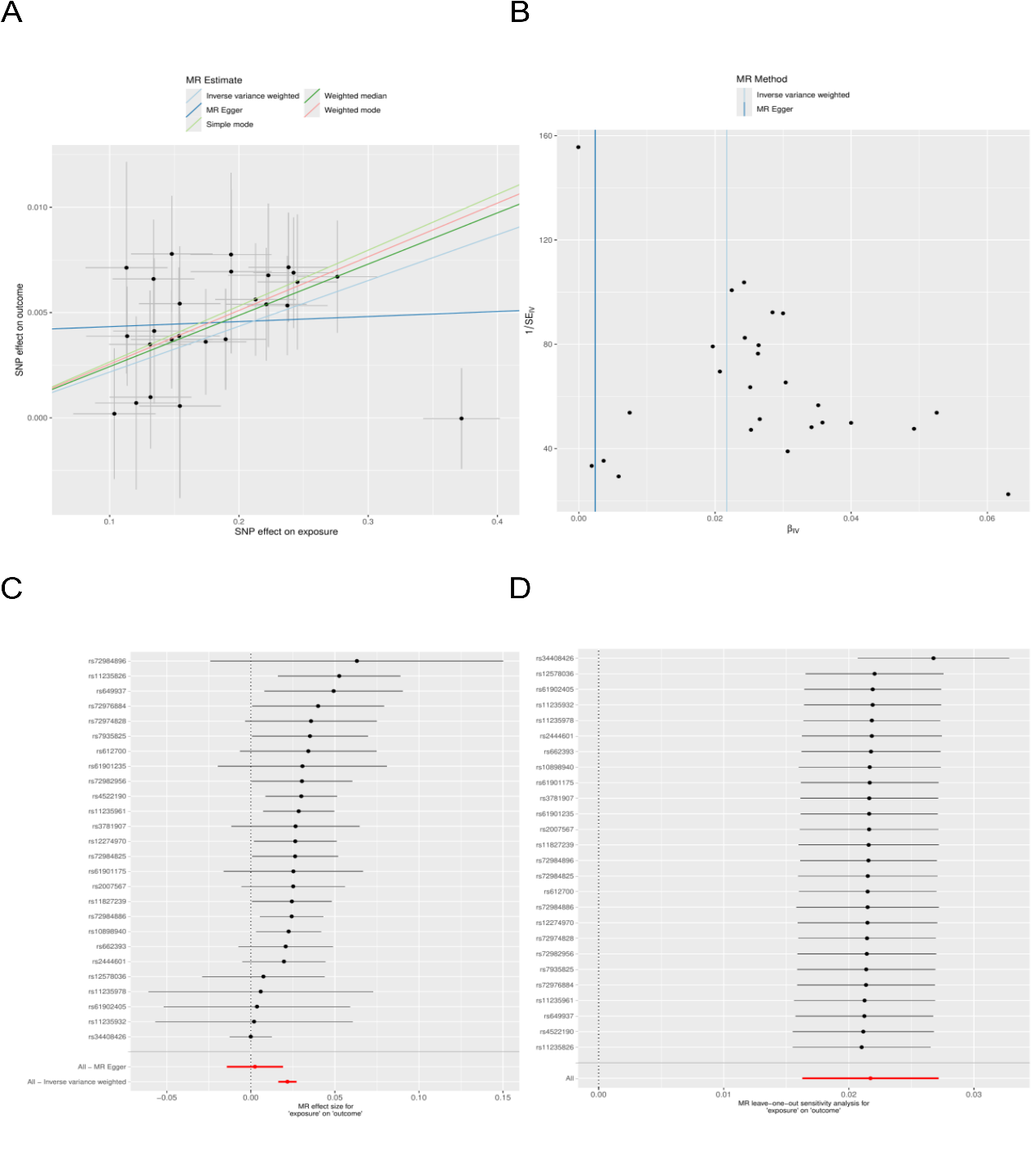
**

**Supplementary Figure S16. Sensitivity analyses for the causal association between *UCP2* expression in** **nk and MDD risk.**

(A) Scatter plot illustrating the causal effect of *UCP2* expression on MDD. Each point represents a single instrumental SNP. The slope of the regression line for each MR method corresponds to the estimated causal effect.

(B) Funnel plot visualizing the distribution of single-SNP effects against their precision. The symmetrical distribution of SNPs around the summary estimate suggests an absence of directional pleiotropy.

(C) Forest plot showing the causal effect estimated by each individual SNP (Single Nucleotide Polymorphism) alongside the combined estimates from the IVW and MR-Egger methods.

(D) Leave-one-out sensitivity analysis. Each point represents the overall MR estimate after removing that particular SNP from the analysis. The results indicate that no single SNP was overly influential on the final causal estimate.
